# Natural and Synthetic Flavylium Derivatives: Isolation/Synthesis, Characterization and Application

**DOI:** 10.3390/molecules30010090

**Published:** 2024-12-29

**Authors:** Diana-Ionela Dăescu, Iulia Păușescu, Ioana Cristina Benea, Francisc Peter, Anamaria Todea, Federico Zappaterra, Andreea Anda Alexa, Alina Ramona Buzatu

**Affiliations:** 1 Biocatalysis and Green Chemistry Group, Faculty of Industrial Chemistry and Environmental Engineering, University Politehnica Timișoara, Vasile Pârvan 6, 300223 Timișoara, Romania; diana.daescu@student.upt.ro (D.-I.D.); iulia.pausescu@upt.ro (I.P.); ioana.benea@upt.ro (I.C.B.); francisc.peter@upt.ro (F.P.); 2 Research Institute for Renewable Energies—ICER, University Politehnica Timisoara, Gavril Musicescu Str. 138, 300501 Timișoara, Romania; 3 Department of Chemical, Pharmaceutical and Agricultural Sciences, University of Ferrara, Via Luigi Borsari, 46-44121 Ferrara, Italy; 4 Department of Biochemistry and Pharmacology, “Victor Babes” University of Medicine and Pharmacy, Eftimie Murgu Sq. No. 2, 300041 Timisoara, Romania; alexa.anda@umft.ro (A.A.A.); buzatu.ramona@umft.ro (A.R.B.)

**Keywords:** natural derivatives, flavylium dyes characterization, anthocyanins, antioxidant activity, synthetic flavylium derivatives

## Abstract

Given the natural origins of flavylium derivatives, their chemical modifications, and their large potential uses in food, medicine, or green chemistry, the present review is a comprehensive study of flavylium-derived compounds. Several topics such as the green extraction and isolation techniques of flavylium derivatives including their chemical modifications and various characterization tools such as NMR, HPLC, and mass spectrometry are discussed in the review. Furthermore, the use of these derivatives in medicine, food, and materials science is presented, highlighting their relevance and the need for further investigation. Therefore, by examining the advantages and disadvantages of natural and synthetic sources, the review asserts the increased relevance of flavylium-based compounds in active molecules.

## 1. Introduction

Natural and synthetic flavylium derivatives belong to the class of flavonoids, a large family of polyphenolic compounds whose structural variety allows a wide range of applications in various domains, including food and biomedical sciences [1,2,3]. Flavylium compounds include anthocyanins (mono- or diglucosides), anthocyanidins (the aglycons of anthocyanins), and 3-deoxyanthocyanins (without the glucoside in position 3, but holding a glucoside unit in position 5) [4,5].

**Figure 1:**
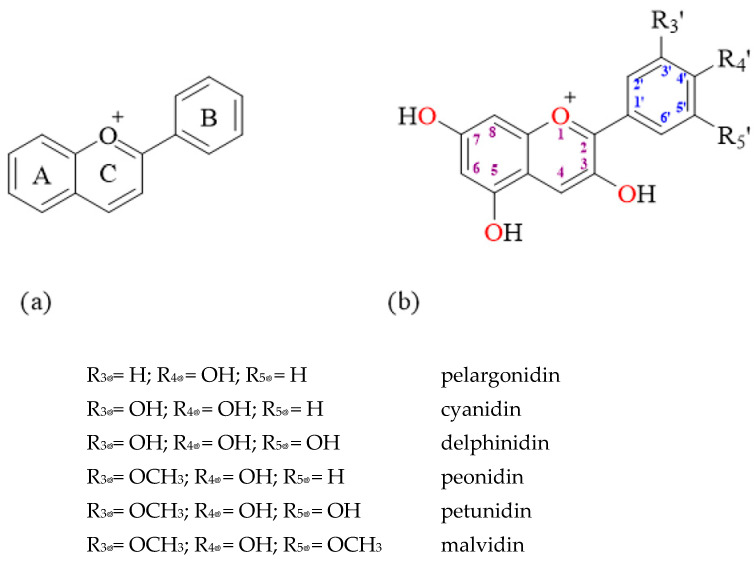
(**a**) The flavylium cation (**b**) Chemical structures of the anthocyanidins most spread in nature (found in more than 90% of the natural anthocyanins).

The flavylium cation ring (AH+), present in anthocyanidins mainly in 7-hydroxylated form, is probably the most versatile natural chromophore. It consists of a positively charged aromatic pyrylium ring (ring C, Figure 1a), supplemented by an additional ring (ring **A**) to create an efficient benzopyrylium conjugated π system. This system seems to be similar to the π systems observed in coumarin and naphthalene. In addition, a single bond stemming from C2 on the electron-deficient pyrylium ring accommodates a benzene ring, leading to an even more extensive conjugation system [6].

The natural anthocyanidins differ from each other in the number of hydroxyl groups attached to ring **B** and their degree of methylation, as well as glycosyl fragments linked at different positions of ring **C** or **A**, which may be acylated with aliphatic or aromatic acids [7].

Anthocyanins are a group of water-soluble plant flavonoid pigments, whose colors range from pink to blue. They are responsible for the color in many fruits and vegetables which makes them one of the most abundant categories of natural pigments in the plant kingdom. Anthocyanins have also been shown to act by repelling herbivores and parasites and defending plants form various stresses. The variations of colors observed in anthocyanins are linked to changes in the surrounding pH environment. As the pH level fluctuates, the structural configuration of anthocyanins suffers modifications, resulting a shift in their observable coloration. In nature, anthocyanins primarily exist as heterozide, while their aglycon forms, known as anthocyanidins, structurally stem from the flavylium (2-phenyl-1- benzopyrylium) cation, exhibiting variations in the positioning of hydroxy and methoxy groups (Figure 1) [8,9,10,11].

Selected examples of representative anthocyanins present in plant sources are shown in Table 1.

Beyond their primary function of pigments for natural dyes, the naturally occurring flavylium derivatives are also responsible for several beneficial effect of the anthocyanins on the food quality or human health, as it will be discussed later in this review. Obviously, the possibility of synthesizing nature-inspired flavylium derivatives, holding the same 2-phenyl-1- benzopyrylium structural unit and different functional groups which could provide improved or new characteristics, attracted considerable scientific interest in the past years. The limited availability of natural anthocyanins was another reason for exploring alternative chemical pathways. Consequently, diverse types of synthetic flavylium derivatives were synthesized, characterized, and evaluated for various applications. Examples of some relevant structures are presented in Table 2. Pyranoanthocyanins, the flavylium derivatives that possess an additional pyranic ring in the anthocyanin structure were included here as well, because apart from the natural forms identified mainly in wines or obtained by microbial methods, they were also synthesized from anthocyanidins or by an entirely chemical synthetic route [16,17].

This review intends to present a comparative overview of recent developments in the synthesis/isolation, characterization, and potential preventive or therapeutic effects of the natural and synthetic flavylium derivatives. In our view it is the first attempt to discuss both categories, allowing us to better understand how nature-inspired research can lead toward structures with different substitution patterns which are able to create additional application possibilities in various fields.

## 2. Isolation of Anthocyanins by Different Extraction Techniques

### 2.1. Extraction of Anthocyanins by Using Organic Solvents

Various solvents are used to extract anthocyanins, including methanol, ethanol, citric acid, water acidified with acetic acid, and ethanol in hydrochloric acid medium [22].

Table 3 provides a detailed overview of the extraction conditions used to recover anthocyanins from different vegetable sources where the key parameters that influence the extraction process, such as solvent composition, temperature, extraction time, and type of plant material used were selected.

Lovelin et al. used a method in which plum fruits were thoroughly cleaned and washed before anthocyanin was extracted using ethanol and citric acid because of its low toxicity. The extraction was carried out using citric acid and ethanol 1 mol/L at a ratio of 80:20 with grounded plum fruit skins. The extraction process involved constant stirring using a magnetic stirrer and was conducted at different times (30, 50, and 80 min) and temperatures (25, 40, and 60 °C). The optimal condition for maximizing the total anthocyanin content was found to be an extraction at a temperature of 40 °C for a duration of 80 min (105.5 mg/100 g) [22].

Zardo et al. performed an extraction of anthocyanins from blueberry pomace which was conducted without the use of organic solvents, as the extract was intended for use in foods. Water, supplemented with 1% *w*/*v* citric acid, was employed as the solvent. The extraction process was carried out in batch mode, using an apparatus consisting of a jacketed cell wrapped in aluminum foil to maintain a constant temperature and shield the solution from light. A thermostatic bath was used for temperature control, and constant stirring was maintained using a magnetic stirrer. Experimental treatments included different combinations of temperature (60 and 80 °C) and time (5, 15, and 45 min), resulting in a total of six treatments. Each batch consisted of 4 g of wet pomace and 60 mL of extraction solution, with a ratio of 1:15 determined to be optimal through preliminary experiments. To evaluate the effectiveness of each extraction, an exhaustive extraction was performed using methanol until no significant staining of the extract was observed. A reduction in the concentration of anthocyanins in the extract was noted when the extraction temperature was set at 80 °C for extended periods. The highest yield was obtained under the extraction conditions of 80 °C for 5 min. In this scenario, 1944.07 ± 46.7 mg of monomeric anthocyanins (specifically, cyanidin-3-glucoside) were obtained per 100 g of pomace on a dry weight basis. This outcome accounts for 81.41% of the total anthocyanin concentration [23].

Patil G. et al. extracted anthocyanins from red radish peels using different extraction mediums. Initially, 50 g of red radish peels were mixed with 100 mL of the chosen extraction medium. The mixture was then ground using a food processor for a standardized duration of three minutes. The solid-liquid ratio was maintained at 1:2 for all extractions. Various extraction mediums were tested, including water, acidified water (1% HCl), different combinations of water and ethanol at varying ratios (ranging from 0 to 80% ethanol), and different combinations of ethanol and acidified water at varying ratios (ranging from 0 to 80% ethanol in acidified water (1% HCl)). It was considered prudent to employ a solution of alcohol in acidified water, with the concentration of HCl maintained at 1%. After extraction, the anthocyanin extract was filtered through muslin cloth to remove any solid particles. It is important to note that only one extraction was performed for each sample to evaluate the efficiency of each extraction medium, with the filter cake being discarded after the initial extraction. The blend of 50% ethanol and acidified water yielded the highest anthocyanin content (37.26 mg/100 mL) [24].

Flores F.P. et al. analyzed the capacity of acetone, ethanol, and methanol to extract anthocyanins from both whole blueberries and pomace. The acetonic extraction involved the use of 70% aqueous acetone solution acidified with 0.01% (*v*/*v*) HCl. The blueberries or pomace were grinded, extracted with acidified acetone, filtered, the residue was re-extracted, extracted with chloroform and finally the aqueous layer was evaporated. For the methanol extraction, blanched whole blueberries were ground with pure methanol acidified with 0.01% (*w*/*v*) citric acid. The third extraction was conducted using freeze-dried blueberry pomace for 24 h with 80% (*v*/*v*) aqueous ethanol solution. For whole blueberries the methanol-based extraction yielded the highest concentration (627.2 mg/L) while the acetone-based extraction produced a substantial yield which was slightly less effective (364.1 mg/L). For pomace the acetone extraction generated a higher yield (982.2 mg/L) compared to ethanol (813.7 mg/L) [25].

Galvão A.C. et al. examined the efficacy of solutions containing water, alcohols, and various organic acids (including citric, adipic, and nicotinic acids) for the extraction of anthocyanins from jabuticaba fruit skins and red cabbage leaves. The experiment involved preparing binary solutions of water with ethanol, water with methanol, and water with isopropanol, each with a molar fraction of 50%. These solutions were created using a semi-analytical scale. Pure solvents and binary solutions were examined both with and without dissolved organic acids. The concentrations of citric and adipic acids were tested at 0.1, 0.5, and 1.0 mol/kg, while nicotinic acid concentrations were assessed at 0.01, 0.03, and 0.06 mol/kg. The highest extraction capacity from jabuticaba skins (36.40 mg/L) was observed using a solution containing water, methanol, and citric acid. Similarly, for the extraction from red cabbage, the highest extraction capacity (56.80 mg/L) was observed using a solution composed of water, methanol, and adipic acid. However, despite yielding the best results, the use of methanol containing solution is not recommended. As alternative ethanol solution was used. For the extraction of anthocyanins from jabuticaba skins, the solution comprising water, ethanol, and citric acid and the obtained yield was 32.06 mg/L of total monomeric anthocyanins. Similarly, for cabbage leaves, the solution of water, ethanol, and adipic acid resulted in a value of 53.82 mg/L of total monomeric anthocyanins [26].

Chatterjee D. et al. aimed to optimize the solvent system composition by incorporating Generally Recognized as Safe status reagents such as water, ethanol, and citric acid. In order to obtain aqueous extracts of food-grade colors from eggplant peels, various parameters were manipulated, including the amount of citric acid, the ratio of water to ethanol, solvent to peels ratio, rotary shaker speed (100, 150, and 180 rpm), and extraction temperature (ambient, 40 °C, 60 °C, and 80 °C) to achieve the optimal yield of color from the peels. Following extraction, the extracts underwent centrifugation for 5 min at 4 °C. Subsequently, the extracts were concentrated using a rotavac system at 40–45 °C and 0.005 MPaHg, and finally, by purging with a gentle stream of nitrogen. The filtered extracts were stored at 4 °C until analysis. At a temperature of 60 °C the researchers obtained a total anthocyanin content of 19.75 g/kg [27].

Kowalska et al. searched for an alternative method to extract anthocyanins that was cheap, easy to apply, and which can be used in food products without raising a concern about food safety. They found that glycerol, possessing lower polarity compared to water, serves as a viable substitute extraction solvent when compared to ethanol. The results indicate that the most efficient extraction of anthocyanins from black chokeberry and elderberry fruits was achieved using water-glycerol extraction systems. Specifically, optimal efficiency was observed with glycerol concentrations of 50% at extraction temperatures of 20 °C and 50 °C, as well as a glycerol concentration of 65% at an extraction temperature of 80 °C. These conditions were identified as yielding the highest levels of anthocyanin extraction from the fruits, highlighting the effectiveness of glycerol-based extraction systems in this process. The results demonstrated that glycerol-water extracts from elderberry fruits exhibited a comparable concentration of anthocyanins to extracts obtained using 50% ethanol [28].

Zannou et al. investigated the efficacy of extracting anthocyanins from borage flowers utilizing a green solvent system composed of choline chloride and glycerol, natural deep eutectic solvents (NADES). The analysis of borage extracts revealed the presence of four distinct anthocyanins: cyanidin-3-glucoside, cyanin chloride, cyanidin-3-rutinoside, and pelargonidin-3-glucoside [29].

Asni H. et al. wanted to apply the eutectic solvent potassium carbonate (K_2_CO_3_)-glycerol to extract anthocyanin pigments from mangosteen peel. The extraction process commences with the preparation of raw mangosteen peel materials, wherein fresh mangosteen peel is carefully selected and washed with clean water. Subsequently, the mangosteen skin is drained, cut into small pieces, and dried in an oven at 50 °C for 12 h. Following drying, the mangosteen skin is ground until it achieves a smooth consistency. Next, the extraction is performed as follows: the extraction equipment is set up, and the mangosteen peel is placed into a three-necked flask. Eutectic solvent is added in a specific ratio, with the ratio of mangosteen peel to eutectic solvent at different values (1:4, 1:5, 1:6, 1:7, 1:8). The extraction takes place for 120 min. After extraction, the mixture is filtered using filter paper to separate the filtrate from the residue. The extracted filtrate is then concentrated using a rotary vacuum evaporator. This extraction procedure is repeated for subsequent treatments. The best results were obtained when using a molar ratio of K_2_CO_3_:Glycerol of 1:7 with a ratio of mangosteen peel: eutectic solvent of 1:8; the anthocyanin content was 263.976 mg/L [30].

### 2.2. Ultrasound Assisted Anthocyanin Extraction

The ultrasound assisted extraction can enhance the extraction of anthocyanins compared to the simple solvent extraction method, in a shorter extraction time. However, the mechanical effects and cavitation generated by ultrasound during the process can potentially damage the anthocyanin structure. Therefore, is crucial to monitor the ultrasonic conditions [31,32].

Xu H. et al. used ultrasound-assisted extraction to enhance the extraction of anthocyanins from purple-fleshed potatoes. This method involved breaking the potato cells using ultrasonic cavitation, which disrupted the cell wall and released the cytoplasm containing anthocyanins. The researchers conducted single-factor experiments to optimize the ethanol extraction concentration and determine the best extracting agent, solid-liquid ratio, and ultrasound time. The ultrasound-assisted extraction method achieved a relatively high anthocyanin extraction rate from purple-fleshed potatoes when the ultrasound time was set at 15 min (0.36% extraction rate). Shorter ultrasound times led to incomplete extraction and lower extraction rates. Conversely, longer ultrasound times beyond 15 min resulted in a gradual decrease in anthocyanin extraction rates [33].

Hu A.-J. et al. investigated the extraction of anthocyanins from blueberry pomace using multi-frequency ultrasonication, with a specific focus on evaluating the impact of ultrasonic power, temperature, and extraction time. In this study, the extraction process was carried out at different ultrasonic powers (250 W, 350 W, 450 W, 550 W, and 650 W) for 40 min at 50 °C using ethanol (60% *v*/*v*) at the solid-liquid ratio of 1:20 g/mL as extraction solvent and two different frequencies: single and dual-frequency ultrasound (80 kHz and 40 + 80 kHz). The maximum anthocyanin yield (10.78 mg/g) was attained using dual-frequency ultrasound (40 kHz and 80 kHz), with an ultrasonic power of 350 W [34].

Zhang S. et al. performed an ultrasound-assisted to improve the anthocyanin extraction from *Vitis davidii* Foex. Pomace using natural deep eutectic solvents (choline chloride-glycerol) at different liquid-solid ratios (10–60 mL/g), ultrasonic times (20–60 min), temperatures (303.15–353.15 K), and ultrasonic powers (280–630 W). The highest total anthocyanin content (3.682 ± 0.051 mg/g) yield was obtained using a liquid-solid ratio of 34 mL/g, an ultrasonic power of 430 W at a temperature of 323.15 K [35].

Devi L.M. et al. aimed to study the effect of ultrasound-assisted extraction factors on total phenolics, flavonoids, and anthocyanin content, with the additional objective of optimizing the extraction process. The extract was obtained by dissolving 1 g of black rice bran in a solution of 25 mL of acidified ethanol (comprising 70% ethanol and 1 M hydrochloric acid). The solution was subjected to sonication at various combinations between time (from 20 to 60 min) and ultrasonic power (from 100 to 300 W). Following sonication, the solution underwent homogenization using a magnetic stirrer for 15 min. Subsequently, centrifugation was conducted at 4500 rpm for 15 min to obtain the supernatant. The best parameters for obtaining the highest total anthocyanin content (11.27 mg cyanidin-3-glucoside/L) were 40 min at 200 W [36].

### 2.3. Microwave Assisted Anthocyanin Extraction

Microwave assisted extraction is the process of extracting chemicals or bioactive compounds such as essential oils and flavonoids from plants, herbs, and other natural sources by quickly heating the solvent in contact with the sample using microwave radiation to facilitate the extraction of specific compounds from the solvent. It is necessary that the selected solvent to be capable of dissolving the compound of interest. One of the significant advantages of MAE is the using of short processing times leading to reducing energy consumption and lower manufacturing costs [37]. Table 4 includes the conditions applied for microwave-assisted extraction (MAE) of anthocyanins from various matrices.

Sun Y. et al. proceeded a microwave-assisted extraction on red raspberries. Frozen fruit samples (60 ± 0.5 g) were processed by crushing them into pieces. These pieces were then placed into a double-neck flask equipped with a cooling system to cool them at room temperature. The flask was filled with an appropriate volume of extraction solvent, consisting of 1.5 M HCl and 95% ethanol (in a ratio of 15:85). This sample was then subjected to microwave extraction using a microwave extractor. The resulting solution was then filtered through paper under vacuum, with the filtrate collected in a volumetric flask. Any residue remaining in the flask was subjected to a second extraction under the same conditions to ensure maximum extraction efficiency. When microwave-assisted extraction was performed with a solvent-to-sample ratio of 4:1 for a duration of 12.1 min at 366 W, the extraction efficiency for total anthocyanins reached 43.42 mg/100 g extract. Eight different types of anthocyanins were identified: cyanidin-3-sophoroside, cyanidin-3-(2-glucosylrutinoside), cyanidin-3-sambubioside, cyanidin-3-rutinoside, cyanidin-3-xylosylrutinoside, cyanidin-3-glucoside [38].

Grigoras C. et al. performed a solvent free microwave assisted extraction of anthocyanins procedure from sweet cherries. Approximately 50 g of fresh fruits were placed in a 250 mL glass vessel without any solvent and exposed to microwave irradiation at 1000 W for four cycles, each lasting 45 s. After each irradiation cycle, the extracted juice accumulated in the vessel was collected in a phial. The combined extracts from the four cycles constitute the crude extract, which was then centrifuged at 7000 rpm for 5 min at 10 °C. The resulting supernatant was promptly frozen at −80 °C to facilitate subsequent lyophilization. This process yielded a red, thin layer of dry extract. The anthocyanins found in sweet cherries were 30 mg/100 g of fresh fruit cyanidin-3-*O*-glucoside and 60 mg/100 g of fresh fruit cyanidin-3-*O*-rutinoside [39].

Duan W. et al. applied microwave-assisted extraction to bring out antioxidant compounds from Chinese bayberry. 0.50 g of bayberry powder was put in a tube then it was blended with an extraction solvent, 1% HCl in 95% ethanol (solid to liquid ratio 1:50). The sample was mixed and irradiated with a power of 800 W, an 80 °C temperature for 15 min (ideal extraction conditions). The utmost yield of total anthocyanins obtained was 2.95 ± 0.08 mg/g [40].

Lutsi N. et al. optimized the anthocyanin extraction process from cocoa shell waste using microwave-assisted extraction. The most favorable conditions were a particle size of 0.105 mm in the extraction, an extraction time of 2 min, and a microwave power 100 W in citric acid:distilled water. The optimal response value for anthocyanin concentration was 11.85 × 10^−4^ M [41].

Evitasari R.T. performed a natural deep eutectic solvent (NADES) microwave-assisted extraction of anthocyanin content in purple sweet potatoes (*Ipomoea batatas* L.) using different material to solvent ratio, microwave power, and extraction time. The deep eutectic solvent was obtained by reacting citric acid and ethylene glycol with a mole ratio of 1:4. The optimum operating conditions were: solid to solvent ratio of 1:29 (*w*/*v*), 270 W microwave power for 193 s, with an optimum total anthocyanin content value of 311.64 mg/L [42].

Alchera F. et al. proceeded with a microwave-assisted extraction using sugar-based NADES (glucose:glycerol:lactic acid in a molar ratio of 1:2:5) as solvent mainly from blueberry peels. A 2 g lyophilized sample was mixed with the correct volume of NADES in a glass vial and then in a high-pressure digestor which can control the temperature. The optimized extraction conditions were determined as follows: the power of microwave of 800 W, extraction heat of 60 °C and the extraction time of 30 min. These conditions resulted in a total anthocyanins content of 634.71 mg/100 g of extract [43].

### 2.4. Anthocyanin Extraction in the Presence of Enzymes

**Figure 2:**
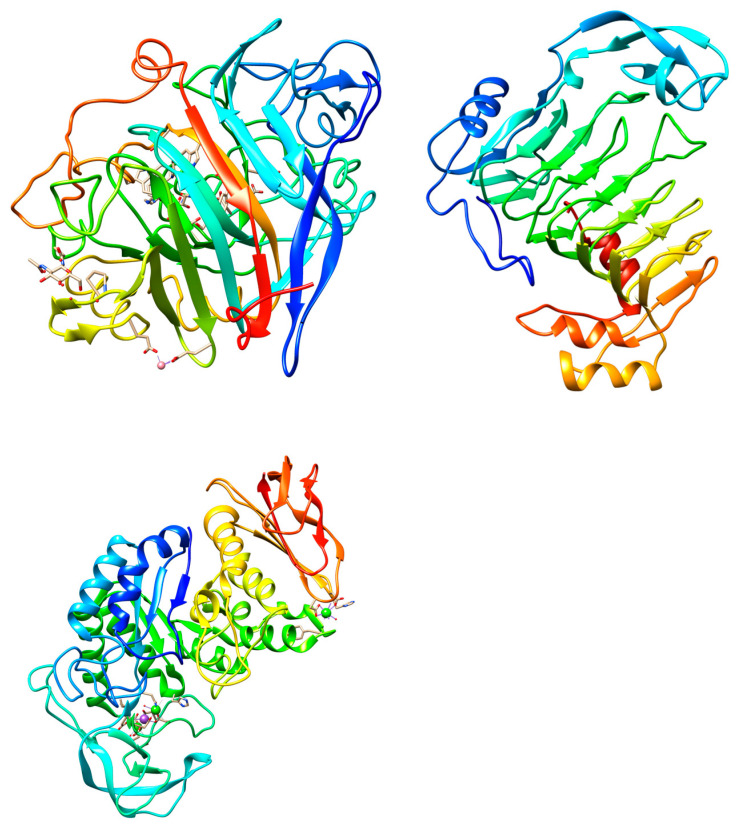
3D structures of cellobiohydrolase from *Trichoderma reesei* (PDB: 6GRN) [47], pectinase from *Aspergillus niger* (PDB: 1QCX) [48], and alpha-amylase from *Bacillus licheniformis* (PDB:1OB0) [49]. The enzyme structures were download from protein data bank and the figures were obtained using PyMOL 2.5.4 software.

Enzyme-assisted extraction of plant bioactive compounds provides multiple advantages, including mild conditions (lower temperature and pH levels), avoiding flammable, volatile, or hazardous solvents, and high specificity for substrates. The aim of using enzymes is to break down the cell wall components of the plant material, which facilitates the release of anthocyanins and other bioactive compounds making the process more efficient and to facilitate the compatibility with purification process. The mild extraction conditions minimize the degradation of sensitive compounds such as anthocyanins preserving their antioxidant properties [44]. Some examples of efficient enzymes and extraction conditions are included in Table 5 and the 3D structures of the three most relevant are presented in Figure 2.

Cell wall degrading enzymes, such as cellulases, pectinases, proteases, xylanases, β-glucosidases, and α-amylases can effectively break down the cell structure, leading to improved extraction of intracellular contents [45,46].

Tran C.-T. et al. studied an enzyme-assisted extraction to produce anthocyanin pigment from purple sweet potatoes. Alpha-amylase was added to dried purple sweet potato powder at a ratio of 1% enzyme to powder weight. The mixture was then incubated for 60 min in a thermostatically controlled shake incubator set at 65 °C and pH 4. Subsequently, anthocyanins were extracted in a water-ethanol mixed solution with a volume ratio of 40/60 mL/mL. The extraction conditions were optimized by varying extraction time (30–70 min) and temperature (5–70 °C). The ideal extraction conditions were determined to be at a temperature of 65 °C and for a duration of 60 min with an obtained anthocyanin concentration of 165 mg/L [46].

Jiang Y. et al. tried to develop a technique, known as radio frequency-assisted enzymatic extraction for extracting anthocyanins from *A. trifoliata* flowers using both the Plackett–Burman (PBD) and Box–Behnken (BBD) experimental designs. The best extraction conditions involved using 0.1% each of cellulase and pectinase (based on the weight of *A. trifoliata* powder), maintaining a pH of 4, using 50% ethanol, having a liquid-solid ratio of 20 mL/g, with a 5 cm gap between radio frequency electrodes, for a duration of 10 min at 40 °C. These conditions resulted in a crude yield of 26.55% and an anthocyanin content of 50.87 mg cyanidin-3-*O*-glucoside (C3G) equivalents/100 g of powder [50].

Roy et al. described the extraction process of anthocyanins making use of *Zymomonas mobilis* to produce ethanol via the Entner–Doudoroff pathway. Utilizing *Zymomonas mobilis* for fermenting grapes offers several notable advantages compared to *Saccharomyces cerevisiae* in maximizing anthocyanin extraction. The organism demonstrates resilience to elevated sugar concentrations, possesses a heightened tolerance to osmotic stress, thrives in environments with increased ethanol levels, and sustains viability in lower pH conditions. Moreover, the fermentation process with *Zymomonas mobilis* does not necessitate strict oxygen control, streamlining its industrial applications. The best recovery of anthocyanins (delphinidin, cyanidin, petunidin, peonidin, and malvidin), 77 mg, was obtained at a fermentation time of 50 h [51].

Amulya et al. optimized enzyme-assisted extraction of anthocyanins from eggplant peel to enhance the recovery of anthocyanins using cellulase enzyme using different temperatures, enzyme concentrations, and time durations. The optimal conditions were 37.32 °C, 5% enzyme concentration, and 1 h in duration resulting in a total anthocyanin content of 578.665 mg C3G/L. This approach not only improved antioxidant activity but also increased anthocyanin yield in comparison with conventional solvent extraction [45].

Swer et al. studied a cellulase-assisted extraction of anthocyanins from Sohiong fruit at five different values of temperature, enzyme concentration, and extraction time. The best level of total anthocyanin content, 9.89 ± 0.24 mg C3G/g, was achieved at 45.6 °C, 12.9% enzyme concentration for an extraction time of 4 h [52].

Lotfi et al. used an aqueous solution of Pectinex at different concentrations, extraction time, and 40 °C to extract anthocyanins from saffron tepals using enzyme-assisted extraction. The optimal conditions to obtain 6.7 mg/g anthocyanins were a temperature of 40 °C, pH of 3.5, extraction time of 60 min, and 5% enzyme concentration [53].

Li et al. used the enzyme-assisted extraction of anthocyanins from the residue of mulberry wine using pectinase enzyme and optimized the extraction process through response surface method testing different liquid to material ratios, pH values, temperatures, and extraction time. The best conditions, which led to an anthocyanin content of 6.04 mg/g, were a liquid to material ratio was 1:20, extraction time was 58 min, pH was 5.9, and extraction temperature was 45 °C [54].

Tecucianu et al. applied an enzyme-assisted extraction on oven-dried and freeze-dried red cabbage using cellulase and pectinase. They used 0.2% cellulase in acetate buffer (pH 4.5) at 40 °C or pectinase in acetate buffer (pH 5) at 55 °C with a solid to enzyme ratio of 1:7 for three incubation times. In the meantime, a combination of equal volumes of cellulase and pectinase in acetate buffer (pH 4.7) at 47 °C for 180 min was performed. The best total anthocyanin content, 708 ± 8.5 mg/100 g dry weight, was attained using both cellulase and pectinase for freeze-dried cabbage [55].

**Table 5:** Examples of enzymes used for anthocyanin extraction and the working conditions.

Source of Anthocyanins	Enzyme	Source of Enzyme	Reaction pH	Total Anthocyanin Content	Reference
Purple sweet potato	α-amylase	*Bacillus licheniformis*	4	165 mg/L	[46]
*A. trifoliata* flowers	Cellulase and pectinase	*Trichoderma reesei* and *Aspergillus niger*	4	50.87 mg C3G/100 g	[50]
Saffron tepals	Pectinex (cellulase, pectinase, and hemicellulose)	*Aspergillus aculeatus*	3.5	6.7 mg/g	[53]
Red cabbage	Cellulase and pectinase	*Aspergillus niger* and *Aspergillus aculeatus*	4.7	708 ± 8.5 mg/100 g	[55]

### 2.5. Supercritical CO_2_ Assisted Extraction of Anthocyanins

Supercritical fluid extraction (SFE) is a separation method based on the use of supercritical fluids instead of organic solvents to extract desired components from solid materials. Due to the unique properties of supercritical fluids, which can penetrate plant materials more effectively, higher yields of anthocyanins can be achieved. Carbon dioxide (CO_2_) has been widely used in food applications due to its advantages including mild critical conditions (31.1 °C and 73.8 bar), non-explosive, non-flammable, non-toxic, available in pure form, cheap and relatively inert to several media [56]. Tested conditions for supercritical CO_2_ assisted extraction of anthocyanins from different matrices were included in Table 6.

Rizkiyah N. et al. used a combination of supercritical carbon dioxide to rip and open the top layer pore of *Hibiscus sabdariffa* (Roselle) and subcritical water extraction to extract anthocyanins from raw material. For the first stage, supercritical CO_2_ extraction, roselle powder weighing 3 ± 0.005 g was added to the extraction vessel. The chiller temperature was set to 6 °C, and extraction proceeded for 60 min. The back-pressure regulator’s heater maintained a temperature of 50 °C. Following CO_2_ pump activation, ethanol was introduced into the system at a rate of 0.24 mL/min (VEtoh/VCO_2_). Pressure was adjusted according to the back pressure regulator, while flow rate was managed by a CO_2_ pump, and temperature was maintained using an oven. The second step used the leftover roselle residue, weighing 200 ± 5 mg, previously obtained sample was transferred to an extraction vessel. The extraction proceeded for 5 min. Pressure adjustments were made using the back pressure regulator, flow rate was managed by a water pump, and temperature was controlled with an oven. For the first stage, the ideal parameters were 20 MPa pressure, 40 °C temperature, and a flow rate of 4.875 mL/min. This configuration led to a total phenolic content of 93.55 mg/100 g. Regarding subcritical water extraction, the optimal conditions were found to be 12 MPa pressure, 140 °C temperature, and a flow rate of 8 mL/min. Under these settings a total anthocyanin content of 1142.61 mg/100 g was achieved [57].

Chatterjee D. et al. aimed to study supercritical carbon dioxide, for extracting color from eggplant peel. For extraction, the peels were pre-treated with a 10% citric acid solution for 1 min before being loaded into the extraction vessel. Testing parameters included extraction pressure (10, 12, 15 MPa), temperature (60, 80 °C), time (90 min–60 min static time plus 30 min dynamic time), and a CO_2_ flow rate of 2 L/min. The most effective conditions determined for the studied extraction to obtain high anthocyanin content (17.04 g/kg dry weight of peel) from eggplant peels were a temperature of 60 °C and a pressure of 10 MPa [27].

Jiao G. et al. used supercritical CO_2_ in conjunction with water as a co-solvent in order to investigate how pressure, temperature, and extraction time affect the yield of total anthocyanin extraction from haskap berry pulp. The extraction process consisted of two phases: static and dynamic. During the static phase, the temperature and pressure were stabilized. Once the desired time elapsed, the static/dynamic valve was opened, and the restrictor valve was adjusted to allow supercritical carbon dioxide to pass through the extraction vessel at approximately 10 mL/min, marking the dynamic stage. The extract was collected in a vial, and CO_2_ was released through a flow meter. After extraction, when the pressure dropped to 0 MPa and the temperature decreased to room temperature, residual berry samples were removed from the vessel, and methanol was used to collect the remaining extract. The washing solution was combined with the initially collected extract, constituting the total extract for the experiment [58].

To ensure careful extraction, the static and dynamic stages were repeated five times. Water was initially added along with the starting materials in the extraction vessel. For the second to fifth extractions, water was introduced into the vessel by the co-solvent pump at a rate of 2.7 mL/min for 2 min without opening the system. The best yield of total anthocyanins, 52.7%, was realized under the conditions of 45 MPa pressure, 65 °C temperature, a ratio of 5.4 g of water to 3.2 g of paste, along with a static time of 15 min and a dynamic time of 20 min [58].

## 3. Chemical Synthesis Pathways of Bio-Inspired Flavylium Compounds

The chemical synthesis of flavylium derivatives related to the anthocyanidins was comprehensively reviewed by Cruz et al. [59]. Therefore, only a short presentation of the main methods will be performed here to provide a more informal overview of the synthetic steps and the possibility to select the best one for the synthesis of similar structures. The most relevant methods reported in the literature for the chemical synthesis of flavylium derivatives are presented in the following sections.

### 3.1. The Algar-Flynn-Oyamada Method

**Scheme 1:**
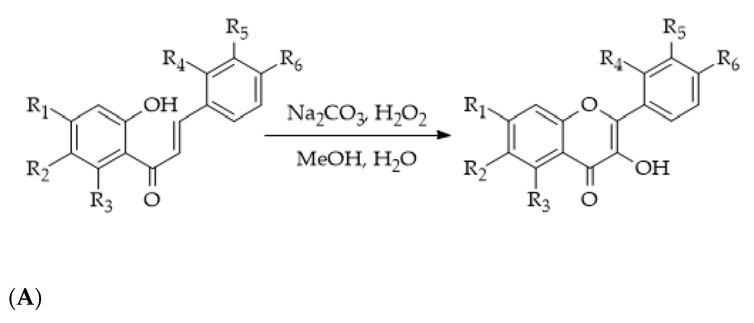

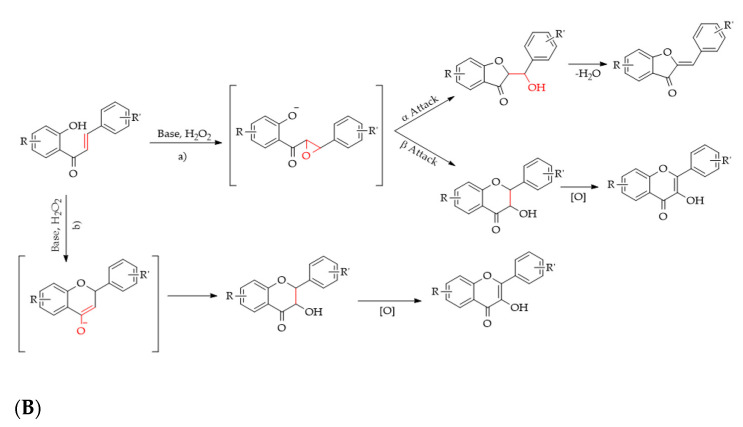
(**A**) Synthesis of 5-substituted flavonols from hydroxychalcones using the Algar-Flynn-Oyamada method. (**B**) Algar-Flynn-Oyamada reaction’s mechanism.

This method involves the oxidation of 2′-hydroxychalcone with hydrogen peroxide in alkaline alcohol solution. However, the reported reaction yields of 5-substituted flavonols were low [60]. As an alternative, Shen et al. used sodium carbonate with hydrogen peroxide to obtain a series (14) of 5- substituted flavonols with moderate to high yields. In terms of reaction conditions, it has been concluded that a combination of methanol/water in a ratio of 2:1 was the most effective reaction medium, and the mixture of 5 eq. sodium carbonate and 2.5 eq. hydrogen peroxide (for 1 eq. chalcone) proved to be the best base. The highest yield of 86% was obtained when R_2_ = R_3_ = R_4_ = R_5_ = H, R_1_ = R_6_ = OCH_3_ (Scheme 1A) [61].

Two hypotheses have been proposed for the mechanism underlying the AFO reaction (Scheme 1B).

The first hypothesis suggests a sequence in which 2-hydroxychalcone is converted to chalcone epoxide. Subsequent cyclization of the epoxide could take place at either the alpha or beta position, resulting in the formation of aurone or flavonol, respectively. The alternative hypothesis for flavonol formation begins with the cyclization of the chalcone anion, followed by the electrophilic attack of hydrogen peroxide at the C-3 carbon atom of the anion, leading to further flavonol formation. Alternatively, these two steps may be combined in a concerted process [61].

### 3.2. Flavylium Compounds Synthesis by Condensation

**Scheme 2:**
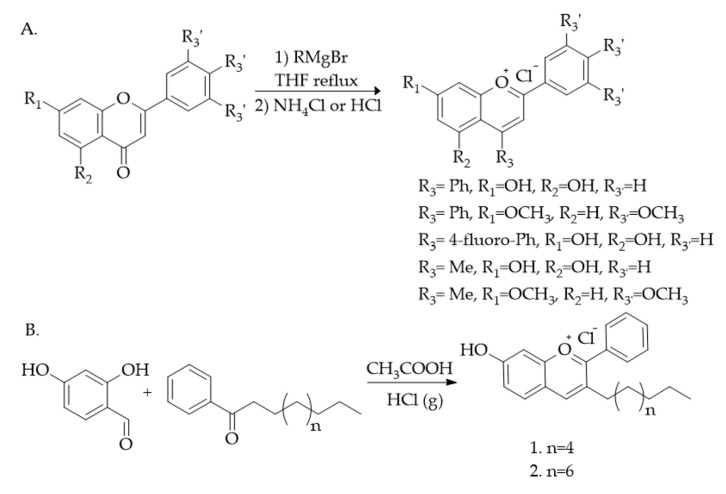

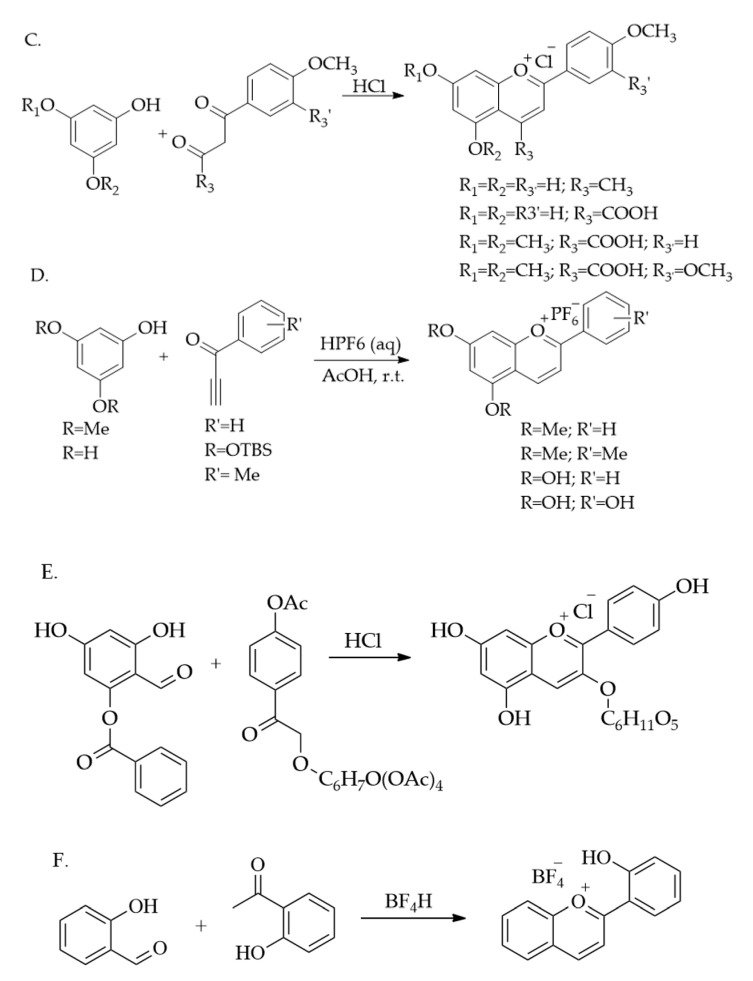
The main routes for the chemical synthesis of flavylium derivatives by condensation reactions. (**A**). Roehri-Stoeckel’s synthesis; (**B**). Fernandes’s synthesis; (**C**). Bülow’s synthesis; (**D**). Kueny-Stotz’s synthesis; (**E**). Robertson’s synthesis; (**F**). Petrov’s synthesis.

A convenient approach to the synthesis of 4-ethoxyflavylium salts involves a one-step acid condensation of *o*-hydroxyacetophenone and its derivatives with aromatic aldehydes and ethyl orthoformate in the presence of 70% perchloric acid. Alternatively, these salts can be prepared by cyclodehydration of *o*-hydroxychalcones with ethyl orthoformate and perchloric acid [62]. The main routes for chemical synthesis of flavylium derivatives by condensation reactions are presented in Scheme 2.

Yakovenko V. I. et al. performed a one-step condensation of 5-bromoresacetophenone with the corresponding aromatic aldehydes and ethyl orthoformate in the presence of 70% perchloric acid. This method directly yields 4-ethoxy-6-bromo-7-hydroxyflavylium salts [62].

Two main synthetic approaches have been widely used to obtain flavylium compounds: the first approach involves the condensation reaction of 2-hydroxybenzaldehydes with acetophenones and the second approach utilizes reductive or oxidative reagents, which are applied to various types of flavonoid compounds [63].

Roehri-Stoeckel C. et al. utilized as source components flavones and Grignard reagents (Scheme 2 route A). These compounds can undergo a reaction resulting in the formation of 4-substituted flavylium salts. In comparison to common 3-deoxyanthocyanidins (aglycones), 4-substituted flavylium salts exhibit characteristic hypsochromic spectral shifts. Additionally, a strong absorption is observed in the near-UV range, typically between 370–390 nm [63].

For the second path, the condensation reactions of 4-N,N-dimethylaminobenzaldehyde and 4-N,N-dimethylaminocinnamaldehyde were carried out using 2, 3, and 7-hydroxy-4-methylflavylium chloride [63].

Fernandes A. C. et al. described a one-step procedure for synthesizing various flavylium salts with alkyl side chains in their 3-, 4-, 5-, or 6-positions (Scheme 2 route B). The synthesis of compounds, featuring alkyl side chains with eight and ten carbon atoms in the 3-positions of the flavylium salts, was achieved through a reaction between 2,4-dihydroxybenzaldehyde and decanophenone or dodecanophenone. Compound 1 was isolated with a yield of 36%, while compound 2 was isolated with a yield of 43% [64].

The acid-catalyzed condensation of phloroglucinol with benzoylacetone and benzoylpyruvic acid, as demonstrated by Bülow et al. resulted in the formation of the 4-methyl and 4-carboxyl flavylium salts in good yields (Scheme 2 route C). These condensations were also efficiently proceeded with *p*-methoxybenzoyl analogs [65].

Kueny-Stotz et al. found that acid-mediated condensation between phloroglucinol derivatives and arylethynylketone in the presence of HPF_6_ (50% in H_2_O) and AcOH, at room temperature, for 48 h successfully resulted in flavylium hexafluoroposphates (Scheme 2 route D) [66].

Robertson et al. successfully obtained an anthocyanin (Scheme 2 route E) without the loss of the glucose residue using *O*-benzoylphloroglucinaldehyde and a glucoside on an acid-catalyzed aldol condensation [67].

Petrov V. et al. achieved through the condensation of salicylaldehyde with 2′-hydroxyacetophenone the synthesis of 2′-hydroxyflavylium tetrafluoroborate (Scheme 2 route F) After stirring overnight, a red solution formed, and a yellow precipitate emerged. This precipitate was filtered, washed with water, and with diethyl ether before being dried [68].

Araújo P. et al. synthesized six amino-based flavylium dyes through aldolic condensation reaction using 2,4,6-trihydroxybenzaldehyde or 4-diethylaminosalicylaldehyde and different acetophenones: 4-amino–, 4-dimethylamino–, 4-hydroxyl–acetophenone, or p-dimethylstyrylmethylketone [69].

## 4. Analytical Methods for the Characterization of Flavylium Derivatives

### 4.1. Chromatographic Techniques—HPLC and HPLC-MS^(n)^

High-performance liquid chromatography is an essential analytical technique for identification and quantitation of the flavylium compounds in natural extracts (basically plant matrices) which contain several anthocyanins with similar structures that makes their chromatographic separation and identification a difficult task. Therefore, optimized sampling and analysis conditions are necessary, as well as mass spectrometric detection coupled with the regular detector, usually an UV-VIS or diode array detector, based on the absorption of anthocyanins in the wavelength domain between 515 and 540 nm [70]. Alternatively, the anthocyanins identification and quantitative analysis can be achieved by using authentic standards, but this option is limited to only the most common types which are commercially available.

Comparatively, the flavylium derivatives obtained by chemical synthesis are usually single products that can be isolated and purified by common separation methods, and may need chromatographic analysis only for the confirmation of the purity. Their analysis is based mainly on NMR techniques to demonstrate the structure of the synthesized compound, as it will be presented in Section 4.4. However, HPLC analysis (coupled or not with MS) was considered a necessary step in some synthetic approaches for flavylium compounds. Oliveira et al. used HPLC-DAD to determine the purity and stability of synthetic amino-based flavylium compounds employing a reversed-phase C18 column (Agilent) 250 × 4.6 mm, particle size 2.7 μm, at 25 °C, and a gradient eluent system consisting from (A) 1% (*v*/*v*) formic acid in water and (B) 0.5% (*v*/*v*) formic acid in 80% (*v*/*v*) acetonitrile. Sousa et al. synthesized new bio-inspired pyrano-3 deoxyanthocyanins with different substituents, using LC-DAD/ESI-MS (alongside NMR) following the purification by column chromatography for the confirmation of the purity and structure. The HPLC analysis was carried out on a reverse-phase C18 column, 150 × 4.6 mm, 5 μm [71].

Table 7 contains a selection of the main HPLC analysis conditions for various anthocyanins isolated from plants. The aim is to provide an overview of the basic requirements in terms of instrumentation and a quick guide to help selecting the right column and eluent system for similar studies.

The analytical HPLC columns used for the separation of anthocyanins (non-acylated or acylated) were mostly nonpolar reverse-phase columns packed with C18 (also named ODS) octadecyl-bonded silica as stationary phase, having different configurations and particle sizes (between 1.8 and 10 μm, mainly 5 μm), depending on the manufacturer and the targeted resolution. The anthocyanins being polar compounds, polar mobile phases were needed for their separation, usually binary gradient systems which include water. The aqueous component of the mobile phase is supplemented with an acid (about 0.1%), e.g., formic acid, acetic acid, trifluoroacetic acid, or phosphoric acid. The organic component of the mobile phase is usually acetonitrile that may be also supplemented with formic or acetic acid, but methanol can be employed as well. The utilization of UV detection at wavelengths between 516 and 535 nm is a common characteristic, the photodiode array detection systems (PDA, also called DAD) being more sensitive than simple UV detectors, leading to better separation in the case of complex mixtures that can result in overlapping peaks.

Excepting the utilization of suitable standards, the identification of the extracted anthocyanins is not possible by only HPLC analysis, needing a hyphenated technique, particularly liquid chromatography coupled with mass spectroscopy (LC-MS or LC-MS/MS). For the coupled LC-MS systems electrospray ionization is commonly used in positive ionization mode (ESI+). In this way, Yang and Zhai separated and identified six anthocyanins in purple corn cob extracts, based on the molecular ion peak and the fragmentation patterns. The mass spectrometer was equipped with an ion spray interface (ISV = 4400) operated in the positive-ion mode and spectra were recorded in positive ion mode between *m*/*z* 200 and 1200 [77]). An excellent overview of the already validated LC and LC-MS methods for identification and quantification of anthocyanins in fruits and vegetables was recently published by Chandra Singh et al. [79]. The development in this field is amazing, as illustrated by the utilization of ion mobility spectrometry (IMS) as a complementary analytical tool in HPLC-MS systems, increasing the sensitivity and allowing better compound identification in anthocyanin analysis. Thus, Ion Mobility Spectrometry High Resolution Time of Flight Mass Spectrometry (IMS-HRTOF-MS) coupled with UHPLC was used for characterization and quantitation of anthocyanins from tea leaves. Excellent results were recently reported by Schnitker et al. for the analysis of very complex matrices of blueberry and grape varieties using UHPLC-ESI(+)-TIMS-QTOF-HR-MS/MS. Structural data were obtained for 54 structurally related anthocyanidin derivatives including dihexosides, monohexosides, pentosides, acetyl- and cinnamoyl conjugates, and allowing among others the differentiation of 3,5-*O*- from 3,7-*O*-diglycosylated anthocyanins. In the reversed phase chromatographic system (Luna^®^ Omega C18 column, 150 × 2.1 mm, 1.6 μm particle size), for a given sugar moiety and positional isomer, the elution order was delphinidin, cyanidin, petunidin, pelargonidin, peonidin, and malvidin [80].

Because MALDI-TOF mass spectrometry can be specifically useful for the identification of anthocyanins, allowing even quantitative estimations, it will be discussed in a dedicated subsection of this review.

### 4.2. Anthocyanin Analysis by Capillary Electrophoresis

Capillary electrophoresis (CE) is a very versatile separation technique based on the charge-to-mass ratio and electrophoretic mobility of the ions, characterized by short analysis times, reduced solvent consumption and lower costs compared to the hyphenated HPLC-MS systems [81].

Bridle et al. successfully separated anthocyanins from strawberries and elderberries extracts. In the strawberry extract they separated: pelargonidin 3-rutinoside, pelargonidin 3-glucoside, cyanidin 3-glucoside, and pelargonidin 3-succinyl glucoside meanwhile elderberry anthocyanins were: cyanidin 3-sambubioside-5-glucoside, cyanidin 3,5-diglucoside, cyanidin 3-glucoside [82].

Da Costa C. et al. separated the blackcurrant anthocyanins using capillary zone electrophoresis. The separation process involved evaluating fused-silica and polyacrylamide-coated capillary columns to determine their ability to resolve the closely migrating analytes. Optimal qualitative separation was achieved on a fused-silica capillary with a phosphate running buffer containing 30% acetonitrile at an apparent pH of 1.5. Using these parameters allowed for the successful separation of the four major anthocyanins present in blackcurrant juice (cyanidin 3-glucoside, cyanidin 3-rutinoside, delphinidin 3-glucoside, and delphinidin 3-rutinoside) [83].

Saénz-López R. et al. identified anthocyanins in red wine with and without SO_2_ addition. In both situations were identified malvidin-3-*O*-glucoside, cyanidin-3-*O*-glucoside, and peonidin-3-*O*-glucoside [84]. Also, Bednár P. et al. (2005) analyzed wine and must and determined the presence of malvidin-3-*O*-glucoside, malvidin-(6-acetyl)- 3-*O*-glucoside, malvidin-(6-coumaryl)- 3-*O*-glucoside, and petunidin-3-*O*-glucoside [85].

The main drawback of the CE analysis (as in the case of common HPLC systems) is that qualitative identification and quantitative analysis are possible only when appropriate standards are available. However, coupled systems that use a mass spectrometry detector instead of the usual UV-VIS detector can resolve these problems, also increasing the selectivity and sensitivity of the analysis [86]. Electrophoretic separation followed by MS identification of the anthocyanins from *Hibiscus sabdariffa* L. was reported by Segura-Carretero et al. The optimized CE-ESI-TOF-MS conditions were running buffer 200 mM borate-ammonium, pH 9, voltage 25 kV, 20 s injection time, allowing the identification of delphinidin-3-sambubioside and cyanidin-3-sambubioside as main components (sambubiose is the β-d-xylosyl-β-d-glucose disaccharide), as well as cyanidin-3-*O*-rutinoside, delphinidin-3-*O-*glucoside, cyanidin-3,5-diglucoside, and chlorogenic acid as minor components [87].

### 4.3. Characterization of Anthocyanins Using MALDI-TOF Mass Spectrometry

Matrix-assisted laser desorption ionization coupled with time-of-flight mass analyzer (MALDI-TOF) is a powerful mass spectrometry technique having the advantage that complex samples such as extracts can be analyzed directly, without the need for prior purification steps such as HPLC or liquid-liquid extraction. This eliminates the risk of sample loss during pre-treatment [88].

MALDI-MS can play a significant role in providing anthocyanin “fingerprints” to authenticate samples. Due to variations in responses across different solvent systems, the absolute number of anthocyanins in the samples might be misinterpreted when compared to a single external standard. In contrast, MALDI-MS demonstrated a more consistent response for a group of anthocyanins compared to HPLC [89]. However, Petropulos et al. demonstrated that a sample cleanup that requires only few minutes and small volumes of solvents is recommended to be performed before the MALDI-TOF MS analysis, proposing solid-phase extraction with Zip-Tip^®^ C18 pipette tips for better data quality. Using this pretreatment and 2′,4′,6′-trihydroxyacetophenone (THAP) as matrix, a total number of 22 anthocyanins and derived pigments were identified in red wine samples, including 10 anthocyanins [90].

As also shown in the examples presented in Table 8, MALDI-TOF MS can be successfully used for the identification of the anthocyanins present in various plant sources based on the molecular weight determination directly from the ion abundance in the mass spectrum. Different matrices were used, including (2,5-dihydroxybenzoic acid (2,5-DHB), α-cyano-4-hydroxycinnamic acid (CHCA), sinapinic acid (SA) or the above mentioned 2′,4′,6′-trihydroxyacetophenone (THAP). Positive ionization mode is mostly preferred for the acquisition of mass spectrometry data. As possible disadvantages, the ions generated by potential fragmentation of the matrix that can impede the identification of flavonoids with small molecular weights and the impossibility to distinguish between anthocyanins and other flavonoids which generate ions of the same *m*/*z* value, should be considered [70].

### 4.4. Structural Analysis of Natural and Synthetic Flavylium Compounds by NMR Spectroscopy

Due to the structural complexity of flavilium derivatives, two-dimensional (2D) NMR spectroscopy has become an indispensable tool for the structural investigation and study of flavilium derivatives [20,94].

Some of the key 2D NMR techniques such as correlation spectroscopy (COSY), heteronuclear single quantum coherence (HSQC) and heteronuclear multiple bond correlation (HMBC) are mandatory molecular structure validation have been reported as useful techniques for flavylium derivatives characterization [20,94].

The application of these techniques is particularly relevant in distinguishing flavylium derivatives with subtle structural variations, such as differences in glycosylation or acylation patterns. For example, 2D NMR has proven effective in pinpointing glycoside positions and verifying linkages, which are critical for understanding the derivatives chemical behavior, stability, and potential applications in materials science and biochemistry. In addition, by combining data from these techniques, researchers can detect impurities or by-products, ensure the integrity of synthesized derivatives, and guide refinement of synthetic protocols [20,94]. Some relevant examples are mentioned in the following paragraphs.

Reiersen B. et al. confirmed the presence of cyanidin 3-*O*-b-galactopyranoside, cyanidin 3-*O*-(2′′-galloyl-b-galactopyranoside) cyanidin and detected for the first time 3-*O*-(2′′-*O*-galloyl-6′′-*O*-a-rhamnopyranosyl-b-galactopyranoside) in chenille plant, *Acalypha hispida*, while Saito T. et al. (2011) identified for the first time petunidin-(E)-p-coumaroylgalactopyranoside and delphinidin-3-*O*-β-d-(6-(E)-coumaroyl)glucopyranoside in addition to delphinidin-3-*O*-β-d-galactopyranoside, delphinidin-3-*O*-β-d-glucopyranoside, cyanidin-3-*O*-β-d-galactopyranoside, and cyanidin-3-*O*-β-d- glucopyranoside [95,96].

Mateus et al. and Giusti M. et al. highlighted the importance of 1D and 2D NMR techniques to elucidate the structure of anthocyanins in red wines and red radish, also, Acevedo De la Cruz A. et al. used a combination between LC-MS and LC-NMR to separate and determine 33 anthocyanins present in four grape wine species: *Vitis vinifera, Vitis amurensis, Vitis cinerea* and *Vitis X champinii* [97,98,99].

**Figure 3:**
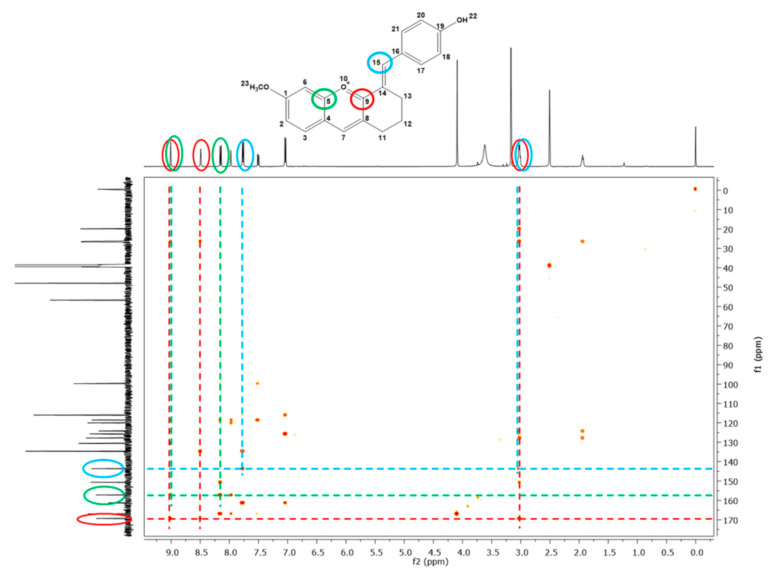
The 1H-13C-HMBC spectrum of a flavylium derivative (4-(4-Hydroxybenzylidene)-6-methoxy-1,2,3,4-tetrahydroxanthylium chloride) synthesized by chemical route—the remote couplings between carbon atoms and protons are depicted by different colored circles, green—C5 with H3 and H7, red—C9 with H7, H11, H13, and H15, blue—C15 with H13, H17, and H21.

Koch et al. confirmed the structure of several derivatives using 2D NMR. For example, the structure of (4-(4-hydroxybenzylidene)-6-methoxy-1,2,3,4-tetrahydroxanthylium chloride) was further supported by the presence of distant couplings between several carbon and proton atoms in the two-dimensional HMBC spectrum (Figure 3).

The signal of the C9 atom, which has a chemical shift of 169.3 ppm, showed two-bond correlation peaks with protons H7, H15, and H11/H13, demonstrating that the carbon atom is indeed bonded in the molecular structure. Similarly, the C5 carbon atom (δC 157.1 ppm) showed the linkage to protons H3-8, which conclusively led to its linkage to H7 at 9.01 ppm and lent credence to the proposed structure. In addition, the remote coupling of the C15 carbon atom (δC 143.6 ppm) was observed to correlate with protons H13, H17, and H21 (7.76 ppm) and confirmed the formation of the desired compound [100].

Păușescu I. et al. recorded chemical shift in ppm versus tetramethylsilane (TMS), which served as a standard reference, and by comparing the observed shifts with those expected for the target flavylium structure, they could confirm if the correct compounds were synthesized. Also, the splitting patterns observed in NMR spectra provided insights into the number of neighboring protons, information that was helpful in verifying if the synthesized compound matched the intended design [20].

To summarize, 2D NMR techniques not only facilitate comprehensive structural analysis of flavylium derivatives, but also serve as a robust tool for advancing our understanding of their chemical properties and interactions. Their role in confirming synthesis success and elucidating structural details underscores their importance in the study of these versatile compounds.

### 4.5. Characterization of Anthocyanins Using UV-Vis Spectroscopy

Generally, the UV-Vis spectrum of anthocyanins shows distinct absorption characteristics. In the visible region, absorption maxima (λ_max_) are typically observed around 510–520 nm, accompanied by a hump in the range of 400–450 nm. Additionally, a peak is often observed in the range of 310–340 nm, which varies depending on the type of anthocyanin and its substitution pattern. The UV-Vis spectra of acylated and non-acylated anthocyanins may differ. A typical UV-Vis spectrum of an anthocyanin shows two main groups of absorbance: one in the wavelength region of 260–280 nm (UV region) and the other in the range of 490–550 nm (visible region). Additionally, when the sugar component is acylated, an additional peak may appear in the 310–340 nm range [101].

The UV-Vis spectrum offers valuable insights into the structural composition of anthocyanins. For instance, the presence of hydroxyl or methoxy groups in the B ring of anthocyanidin influences the color and absorption properties of anthocyanins. Increased hydroxyl substitution leads to a bathochromic shift (red shift), whereas methylation results in a hypsochromic shift (blue shift) in the absorption maxima. It also can help predict the number and type of glycosylation and acylation present in anthocyanins [101].

Integrating UV-Vis spectroscopy with LC-MS enhance the identification and quantification of anthocyanins [101].

Table 9 summarizes the comparison of calculated and measured absorption wavelengths for several compounds within the ultraviolet (UV) spectrum. The electronic absorption spectra were calculated in methanol using the IEFPCM model by time-dependent DFT (TD-DFT) calculations on the optimized structures at the B3LYP/6-31+G(d,p) level of theory, taking into account the lowest six singlet→singlet spin-allowed excited states. It can be seen that the two sets of values, i.e., calculated and measured wavelengths, are generally quite close, but there are some small shifts. These small differences could be the result of factors such as experimental design, instrumental imperfections and/or simplifications of the theoretical approach. Despite these small differences, the results obtained show that the calculated wavelengths are in reasonable agreement with the measured data, which means that the present model can predict the UV absorption characteristics to a reasonable degree. Improvements in computational models and experimental designs may bring the two sets of measured values closer to comparable values than is currently the case.

Liu et al. explored the effects of various processing methods on the anthocyanin composition and antioxidant capacity of blueberry juice using metabolomics and density functional theory (DFT) analysis. The study aimed to identify optimal techniques for preserving anthocyanins, which are known for their antioxidant properties but have poor stability. A total of 54 anthocyanins were identified in the juice, with different treatments (thermal, high pressure, electric and magnetic fields, and cobalt source irradiation) impacting the stability and antioxidant potential of these compounds. The findings indicated that while processing did not alter the types of anthocyanins present, it significantly affected their concentration and antioxidant activity. Notably, the 1 kGy irradiation treatment provided the best protection for anthocyanins, preserving their antioxidant capacity more effectively than other methods. The study also found that the antioxidant capacity of the juice was not solely dependent on the total anthocyanin content but was influenced by the specific composition of anthocyanin monomers. DFT calculations confirmed that anthocyanins such as Pt3Ga and M3Ga had higher antioxidant capacity compared to M3G and Mva. These results highlight the importance of selecting appropriate processing techniques to enhance the antioxidant stability of anthocyanins in blueberry juice, offering insights for improving the quality of anthocyanin-rich beverages [102].

## 5. Preventive or Pharmacological Effects of Flavylium Compounds

Anthocyanins have a variety of benefits on human health as: anti-fatigue [103], anticancer [104,105], antiobesity, and antidiabetic [106], anti-inflammatory, and anti-proliferative effects [107,108], antioxidant activity [109], improving brain functions [110], and reducing cardiovascular diseases [111,112]. PFEA significantly extended weight-loaded swimming time, reduced levels of lactate dehydrogenase (LDH), blood lactate (BLA), and blood urea nitrogen (BUN), while increasing liver glycogen (LG). PFEA could stabilize oxidative stress biomarkers (MDA), antioxidant enzymes (SOD), and inflammatory cytokines (TNF-α, IL-1β, IL-6). Additionally, PFEA up-regulated mRNA expression of PGC-1α and PPARα in skeletal muscles [103].

Kimble et al. investigated the anti-fatigue properties of anthocyanins found in Tart Montmorency cherries (MC), which are notably rich in anthocyanins and other polyphenols known for their antioxidant, anti-inflammatory, and vasomodulatory actions. This study aimed to determine the impact of chronic MC supplementation on cognitive function and mood. In a 3-month double-blinded, placebo-controlled parallel study, middle-aged adults (mean ± SD: 48 ± 6 years) were randomly assigned to either 30 mL twice daily of MC (n = 25) or the same amount of an isoenergetic placebo (n = 25). After 3 months, the MC group exhibited higher accuracy in digit vigilance (mean difference: 3.3, 95% CI: 0.2, 6.4%) with fewer false alarms (mean difference: −1.2, 95% CI: −2.0, −0.4) compared to the placebo group. There was also a treatment effect for higher alertness (mean difference: 5.9, 95% CI: 1.3, 10.5%) and lower mental fatigue ratings (mean difference: −9.5, 95% CI: −16.5, −2.5%) with MC. Plasma metabolomics revealed an increase in several amino acids in response to MC intake, but not placebo. These findings suggest that MC supplementation has anti-fatiguing effects and can improve sustained cognitive performance and mood [113].

### 5.1. Antibacterial Activity

Correia P. et al. investigated the antibacterial activity of some amino-based flavylium dyes, and they demonstrated the ability to inhibit bacterial growth of Gram-positive bacteria (*Staphylococcus aureus* and *Staphylococcus epidermidis*) and Gram-negative bacteria (*Pseudomonas aeruginosa*) being able to manage microbial skin infections [114].

### 5.2. Antiobesity

Salehi et al. studied the anti-obesity and anti-diabetic effects of anthocyanins. Their findings highlighted several mechanisms through which anthocyanins exert these effects. Anthocyanins were found to increase energy expenditure and thermogenesis, upregulating uncoupling proteins (UCP1 and UCP2) in brown and white adipose tissues, respectively. For example, berries containing petunidin (33%) and malvidin (57%) were effective in lowering high-fat diet (HFD)-induced metabolic damage by increasing energy expenditure and reducing mitochondrial respiration and proton leak in adipose tissue. Additionally, anthocyanins modulated AMP-activated protein kinase (AMPK), a key regulator of energy balance, enhancing mitochondrial biogenesis, reducing lipid metabolism, increasing fatty acid oxidation, and improving insulin sensitivity [106].

### 5.3. Antidiabetic

In 2021, Les et al. reviewed the role of anthocyanins as antidiabetic agents, summarizing, in particular, their mechanism of action in human studies [115]. The positive effect of anthocyanins on diabetes is due to the inhibition of several physiological enzymes involved in the regulation of glycaemia, such as α-amylase, α-glucosidase, protein tyrosine phosphatase 1B, and dipeptidyl peptidase IV. For example, a purified extract from black carrots demonstrated the ability to inhibit the enzymes α-amylase, α-GLU, and DPP-IV. The IC50 values for this extract were even lower than those of standard inhibitors such as acarbose and vildagliptin. The primary anthocyanin responsible for this inhibition was Cy 3-xylosyl-galactoside [116]. α-glucosidase (α-GLU) another digestive enzymes in charge of the hydrolysis of carbohydrates and target of pharmacological inhibition by clinical drugs for the treatment of type 2 diabetes, can be inhibited by anthocyanins in blueberry, cranberry, and cherry juices [117,118,119].

In clinical studies, bilberry anthocyanins increased AMP-activated protein kinase (AMPK) activity in skeletal muscle and liver, enhancing glucose uptake and utilization, and reducing liver lipid content and serum lipoproteins. Furthermore, supplementation with a daily intake of four cups of freeze-dried strawberry beverage over 8 weeks in 27 diabetic subjects reduced total and LDL-cholesterol levels and inhibited vascular cell adhesion molecule-1 (VCAM-1) circulating levels [120]. Another study showed that anthocyanins significantly inhibited body weight gain, improved glucose tolerance, enhanced insulin sensitivity, and decreased hepatic lipid accumulation by modulating AMPK activity and lipid metabolism-associated gene expression. These results underscore the potential of anthocyanins in managing obesity and diabetes, promoting weight loss, improving lipid profiles, and enhancing glucose metabolism [106].

### 5.4. Anti-Inflammatory and Anti-Proliferative Effects

Vendrame et al. studied the anti-inflammatory and antiproliferative effects of anthocyanins. Their findings demonstrated that anthocyanins exert significant anti-inflammatory effects by modulating key signaling pathways. Specifically, anthocyanins were shown to downregulate the nuclear factor-κB (NF-κB) signaling pathway, reducing the expression of proinflammatory cytokines such as TNF-α, IL-1β, and IL-6. Additionally, anthocyanins inhibited the mitogen-activated protein kinase (MAPK) pathways, leading to decreased phosphorylation of ERK, JNK, and p38 MAPKs. These effects result in the attenuation of inflammation and proliferation of inflammatory cells. The study concluded that anthocyanins could effectively reduce inflammation and proliferation, suggesting their potential therapeutic role in managing inflammatory diseases [108].

### 5.5. Antioxidant Activity

In this subchapter examples that highlights the antioxidant capacity of anthocyanins, emphasizing their structural features, stability, and applications in diverse contexts were selected. The reviewed studies cover various aspects, including the mechanisms behind their antioxidant effects, the influence of structural modifications on bioactivity, and novel strategies for enhancing their stability and bioavailability in different systems. This compilation highlights recent advancements in the use of anthocyanins in food, cosmetics, and therapeutic applications, with particular focus on their role in combating oxidative stress and supporting overall health.

A defining characteristic of anthocyanins is their exceptionally strong antioxidant capacity, which exceeds that of other flavonoids [121]. Anthocyanins can effectively neutralize harmful free radicals by chelating metal ions and serving as hydrogen donors to reactive oxygen species [122]. The antioxidant potency of anthocyanins is closely tied to their structure. For example, glycosylation and sugar attachment can reduce their antioxidant activity, whereas an increase in the number of hydroxyl groups typically enhances it [123,124].

Tsuda et al. (1996) examined the antioxidative, radical scavenging, and lipid peroxidation inhibitory effects under UV light irradiation of three anthocyanin pigments—pelargonidin 3-*O*-β-d-glucoside (P3G), cyanidin 3-*O*-β-d-glucoside (C3G), and delphinidin 3-O-β-d-glucoside (D3G)—isolated from the Phaseolus vulgaris L. seed coat, as well as their aglycons, pelargonidin chloride (Pel), cyanidin chloride (Cy), and delphinidin chloride (Del). All pigments exhibited strong antioxidative activity in the liposomal system and reduced malondialdehyde formation under UVB irradiation. However, the antioxidative activity in a rat liver microsomal system and the scavenging effects on hydroxyl radicals (·OH) and superoxide anion radicals (O^2−^) varied depending on their structures [109].

Lipophilic anthocyanins with chain lengths of up to C8 demonstrated enhanced antioxidant activity compared to their precursors, providing a stronger protective effect [125].

It has been suggested that the accumulation of anthocyanins in young leaves may act as a natural “sunscreen” by absorbing visible and UV light, thereby safeguarding the plant’s photosynthetic machinery. Additionally, their antioxidant properties could provide further protection to plant cells by mitigating oxidative damage [126,127].

Toxicological studies have demonstrated that anthocyanins are safe for human health and exhibit beneficial effects. The antioxidant and antiproliferative activities of strawberry crude extracts and purified anthocyanins were assessed using the Trolox Equivalent Antioxidant Capacity (TEAC) assay and luminescent ATP cell viability tests. Notably, among the isolated compounds, cyanidin-3-glucoside, pelargonidin, and pelargonidin-3-rutinoside emerged as the most potent antioxidants [128].

Mohammadi et al. recently explored the sustainable extraction and application of berry anthocyanins in functional gummies, comparing the antioxidant properties of blackberry (BB) and elderberry (EB) extracts. The study found that EB extracts consistently exhibited higher total phenolic content, anthocyanin content, and antioxidant capacity than BB extracts. However, over a 120-min period, BB extracts demonstrated a stronger potential to inhibit lipid oxidation in human plasma. Both extracts displayed pH-dependent color shifts and variations in antioxidant capacity, with EB extracts maintaining greater stability across a wider pH range. Freeze-drying effectively preserved antioxidant activity, with EB extracts retaining higher levels than BB. When incorporated into gummy formulations, these extracts resulted in significantly higher phenolic and anthocyanin content compared to commercial gummies, with EB gummies displaying superior antioxidant capacity [129].

Zang et al. conducted a study to enhance the antioxidant activity of anthocyanins from purple potatoes by formulating water-in-oil-in-water (W/O/W) nanoparticles to improve the bioavailability of these compounds. In vivo tests on rats demonstrated a 220.36% increase in anthocyanin absorption, highlighting their potent antioxidant properties and potential health benefits [130].

Jiang et al. recently investigated the effects of superfine grinding on purple whole wheat flour (PWWF), focusing on its impact on the anthocyanin profile, as well as its physicochemical and antioxidant properties. Antioxidant capacity was assessed using three assays: TPC, ABTS, and ORAC. The results indicated that superfine PWWF exhibited up to 1.6 times higher antioxidant capacity compared to the original PWWF [131].

The limitations in the bioavailability of anthocyanins were minimized by Li et al. through microencapsulation with fructooligosaccharides (FOS). The results demonstrated that anthocyanin-loaded microencapsulated particles (ALM) exhibited significantly higher antioxidant capacity compared to free anthocyanins (ANCs) and cyanidin-3-glucoside (C3G). During simulated digestion, ALM showed improved retention of anthocyanins compared to ANCs in both gastric and intestinal phases. This microencapsulation approach using FOS and whey protein (WP) effectively enhanced the antioxidant capacity and stability of anthocyanins during in vitro digestion [132].

Different phenotypes and parts of Japanese maca (*Lepidium meyenii*) were recently studied in terms of their antioxidant activities, as well as their total polyphenol, anthocyanin, and benzyl-glucosinolate content. The study highlights that the purple maca skin exhibited the highest levels of total polyphenols, antioxidant activity, and anthocyanin content among all maca varieties [133].

The antioxidant activity, anthocyanin content, and polyphenol profile of *Ardisia compressa* K. extracts were investigated by Rodríguez-Aguilar et al. who also assessed the effects of time and temperature, as well as the addition of sucrose and citric acid (CA). Extracts with added CA demonstrated the highest levels of total phenolic content (TPC), monomeric anthocyanin content (MAA), and total antioxidant capacity (TAC), as measured by DPPH and ABTS assays [134].

Shiau et al. recently investigated *Clitoria ternatea* flower extracts, rich in phytochemicals and antioxidants, for their potential application in foods and nutraceuticals at neutral and acidic pH levels. The study reported that antioxidant activity was measured by the extract’s capacity to scavenge DPPH (2,2-diphenyl-1-picrylhydrazyl) radicals. Optimal extraction of total anthocyanin content (TAC) and total phenolic content (TPC) was achieved for intact *Clitoria ternatea* flowers (CTF) at 90 °C for 90 min, and for their powdered form (CTFP) at 90 °C for 30 min [135].

Turan Ayseli et al. investigated the effects of gamma irradiation and electrospinning on the physicochemical, antioxidant, and molecular properties of anthocyanin colorants derived from black carrot pomace. The study found that the anthocyanin powder (AP) sample had the highest antioxidant activity, with DPPH and FRAP values of 177.19 mgTE/g and 324.70 mgTE/g, respectively. In contrast, the anthocyanin/gelatin-based nanofibers (NAP), with an AP/gelatin ratio of 1:2, showed the lowest antioxidant activity, with DPPH and FRAP values of 66.26 mgTE/g and 111.80 mgTE/g. The FRAP (Ferric Reducing Antioxidant Power) assay measures the antioxidant effect of a substance by assessing its ability to reduce ferric (Fe^3+^) to ferrous (Fe^2+^) ions, which correlates with antioxidant capacity. These results suggest that irradiation negatively impacted the antioxidant capacity of the AP sample. Measurements for total phenolic content (TPC), total monomeric anthocyanin content (TMAC), and antioxidant activities were consistent across the samples [136].

The study on *Punica granatum* L. (pomegranate) cv. Ghojagh examined how shading and foliar sprays of potassium sulfate and sodium silicate affect fruit quality, particularly focusing on antioxidant traits. Antioxidant properties, including total anthocyanin and phenolic content, are crucial for the quality of pomegranate arils, as they contribute significantly to the fruit’s health benefits and visual appeal. The results indicated that covering the trees with shading nets and applying a 0.15% sodium silicate spray significantly enhanced antioxidant activity and increased levels of superoxide dismutase and catalase—key enzymes in antioxidant defense. At the same time, these treatments reduced polyphenol oxidase and peroxidase activities, enzymes associated with oxidation and browning. The combination of shading and sodium silicate led to the highest total antioxidant activity, promoting higher fruit quality in hot climates by mitigating aril whitening and enhancing the biochemical profile of the fruit [137].

Taghizadeh et al. conducted a study evaluating the phytochemical composition and antioxidant properties of *Centella asiatica* ethanolic extract. The presence of phenolic compounds, including sinapic acid, catechin, quercetin, *p*-coumaric acid, hesperidin, eugenol, and hesperetin, with hesperetin was demonstrated by HPLC and antioxidant properties, attributed to its high phenolic and flavonoid content, placed the extract as potential ingredient for cosmetics and pharmaceuticals. Additionally, the extract demonstrated strong antibacterial effects, particularly against *S. aureus* and *B. cereus*, underscoring its promising role in health-related applications [138].

Ali et al. conducted an evaluation of anthocyanin-enriched wheat varieties (black, blue, and purple) with the goal of enhancing antioxidant content and developing antioxidant-rich biscuits. In this study, 35 advanced wheat lines—spanning white, purple, black, and blue varieties—were assessed for their yield, grain hardness, protein content, dough quality, and biscuit-making attributes. Among these, the black wheat lines (black-1 and black-6) stood out for their high antioxidant activity, which remained 1.3 times greater than the control even after baking, despite some anthocyanin loss. This suggests that the enriched anthocyanin content in these lines contributes significantly to their antioxidant properties. These black wheat lines, which also displayed excellent dough and biscuit quality with soft texture and high spread ratios, highlight substantial commercial potential, offering a promising approach to elevate the nutritional profile of biscuits in the food industry [139].

In a recent study by Simionescu et al. the antioxidant potential of *Weissella confusa* PP29 probiotic media was significantly enhanced through the incorporation of *Hibiscus sabdariffa* L. anthocyanin extract. Known for its robust antioxidant properties, *H. sabdariffa* anthocyanin extract not only protected the lactic acid bacteria (LAB) but also bolstered their development, resulting in a probiotic medium with impressive antioxidant attributes. The study highlighted that the antioxidant capacity of the fermented product—measured via DPPH radical scavenging and FRAP assays—exceeded that of ascorbic acid and typical antioxidant extracts. Furthermore, the product’s superoxide anion radical scavenging and lipid peroxidation inhibition were comparable to ascorbic acid, underscoring the powerful antioxidant effects imparted by the anthocyanin enrichment. Remarkably, these antioxidant properties, closely linked to the initial concentrations of LAB and anthocyanins, were maintained for up to six months, indicating the medium’s potential as a long-lasting, functional food with enhanced health benefits for gut protection [140].

de Moura et al. explored the antioxidant potential of an anthocyanin-rich extract from purple tea (*Camellia sinensis* cv. Zijuan), focusing on its chemical stability, cellular antioxidant activity, and protective effects on human erythrocytes and plasma. The study utilized a sustainable and non-toxic extraction method with an aqueous citric acid solution, enabling the isolation of an anthocyanin extract with robust antioxidant properties. In vitro assays demonstrated that the extract exhibited high antioxidant activity, inhibiting DPPH radicals by 73% at pH 4.5 and 39% at pH 10. Additionally, the extract’s anthocyanins demonstrated considerable thermal stability at 60 °C but showed susceptibility to photodegradation under prolonged light exposure. Advanced analysis via UPLC-ESI-MS and HPLC quantified 33 phenolic compounds, including anthocyanins and catechins, totaling 40.18 mg/g in the lyophilized extract (CLE). The CLE also protected human plasma from oxidative damage (635 ± 30 mg AAE/g) and exhibited cytotoxic and antiproliferative effects against various cancer cell lines, highlighting the extract’s potential for therapeutic and technological applications. These findings underscore the powerful antioxidant capacity of anthocyanin-rich purple tea extract, although careful control of pH, temperature, and light exposure is essential to preserve its bioactivity [141].

A study by Domínguez et al. examined the electrochemical properties of lyophilized blueberry (*Vaccinium corymbosum* L.) and raspberry (*Rubus idaeus* L.) samples, focusing on the activation of antioxidant properties of anthocyanins through reactive oxygen species (ROS). Using microparticulate deposits from ethanolic fruit extracts, researchers observed that electrochemical oxidation of anthocyanins—particularly under ROS generation—produced new species with elevated antioxidant capacity over a wide pH range. ROS-activated anthocyanins demonstrated oxidative loss of sugar moieties and formation of o-quinones from compounds such as cyanidin, pelargonidin, and delphinidin, processes that enhance antioxidant effects. This ROS-induced shift in the antioxidant profile suggests that ROS may serve not only as signal transducers in plant physiology but also as enhancers of anthocyanin antioxidant activity. These findings have significant implications for food chemistry, as they validate that lyophilized fruit samples retain high antioxidant potential and hint at the possibility of using ROS modulation to create anthocyanin-derived compounds with potent antioxidant properties, offering new avenues for food production and preservation [142].

Dong et al. explored the development of innovative nanocomplexes for anthocyanin (ACN) delivery, using ovalbumin (OVA) and sulphated polysaccharides with varying charge densities (κ-, ι-, λ-carrageenan, and dextran sulfate). The study focused on enhancing the stability, bioaccessibility, and antioxidant capacity of anthocyanins through encapsulation in these nanocomplexes. The resulted particles demonstrated high encapsulation efficiency (94.11–96.2%) and loading capacity (9.05–9.54%) for ACNs, with OVA-dextran sulfate (DS) nanocomplexes showing the smallest particle size and greatest stability. These nanocomplexes improved the antioxidant capacity of anthocyanins significantly under accelerated degradation conditions and simulated digestion, particularly for OVA-DS-ACN and OVA-λC-ACN complexes. The study suggests that OVA/sulphated polysaccharide nanocomplexes not only bolster ACN stability and antioxidant properties but also have potential applications as delivery systems in food and nutraceutical industries, offering robust protection and improved bioavailability of hydrophilic bioactive compounds under diverse processing conditions [143].

Wang et al. investigated the impact of thermal processing on the degradation and antioxidant capacity of anthocyanins extracted from purple eggplant peels within a complex food system composed of dietary fiber, protein, and starch. The study revealed that frying at 185 °C led to the most significant reduction in anthocyanin content, with an 84.48% loss, while boiling and steaming treatments resulted in first-order degradation kinetics, and microwave treatments followed second-order kinetics. The antioxidant capacity was found to be positively correlated with the levels of total anthocyanins (TAC) and total polyphenols (TPC). During thermal processing, anthocyanins predominantly broke down into compounds such as gallic acid, phloroglucinaldehyde, and 2,4-dihydroxybenzoic acid, with further isomerization of phloroglucinaldehyde into phloroglucinic acid observed under frying conditions. The correlation coefficients between TPC and antioxidant activity (ABTS, DPPH, and FRAP) were strong, ranging from 0.86 to 0.92, with TAC showing an even stronger correlation with FRAP (0.95). These results indicate that, despite significant degradation, the residual polyphenols—particularly anthocyanins—contribute to the antioxidant properties of eggplant peels after thermal processing, highlighting their potential as valuable antioxidant components in food systems [144].

Akbulut et al. explored the use of black grape pomace, a by-product of fruit juice production, in the creation of shalgam juice, a traditional Turkish fermented beverage. The study aimed to transform this pomace into a high-value ingredient to enrich shalgam juice with phenolic compounds, particularly anthocyanins, tannins, and resveratrol, thus enhancing its antioxidant properties. Five formulations were developed using different proportions of black grape pomace and black carrot, both rich sources of polyphenols. The antioxidant activity (measured via the ABTS assay) increased steadily in samples containing black grape pomace, aligning closely with rising total phenolic content—a pattern that was absent in samples with only black carrot, where antioxidant activity fluctuated. The polyphenolic profile revealed diverse phenolic acids (such as gentisic, caffeic, and ferulic acids), along with resveratrol, rutin, catechin, and various cyanidin glucosides, many of which were uniquely present in shalgam juice formulations containing both black grape pomace and black carrot. This unique combination influenced the antioxidant potential, as the interactions between different polyphenols—some synergistic, others antagonistic—played a role [145].

Zhao et al. investigated the metabolic factors underlying the superior antioxidant capacity of red-fleshed peaches, highlighting their rich phenolic composition. The study revealed that red-fleshed peaches accumulate significantly higher levels of phenolic compounds compared to non-red-fleshed varieties, correlating with a notably stronger antioxidant activity in their crude extracts. Interestingly, the findings showed that total phenolic content, rather than anthocyanins alone, was the primary contributor to the antioxidant effects. Metabolomic analysis demonstrated a coordinated accumulation of anthocyanins, flavonoids, and phenolic acids in red-fleshed peaches, which collectively enhanced their antioxidant properties. These bioactive compounds exhibited dynamic changes throughout fruit development, with both phenolic content and antioxidant activity being more pronounced in the peel than in the flesh. This study underscores the metabolic complexity behind the antioxidant potential of red-fleshed peaches, suggesting their significant health benefits due to their robust phenolic profile [146].

Bao et al. explored the use of sulfated polysaccharides to stabilize and enhance the antioxidant capacity of blueberry anthocyanin (BA) within an oral film matrix. The study utilized chondroitin sulfate (CS), fucoidin (FU), and λ-carrageenan (λ-CG) complexed with BA in hydroxypropyl methylcellulose (HPMC) films, aiming to improve anthocyanin stability, controlled release, and bioactivity. The addition of these sulfated polysaccharides significantly enhanced the retention and stability of BA, particularly in the CS-BA/HPMC system, which maintained a 5.5-fold higher anthocyanin retention after 8 days of light-accelerated storage compared to the control. The study also demonstrated that the BA-infused oral films exhibited superior antioxidant properties, as measured by DPPH and ABTS radical scavenging assays. Notably, the antioxidant activity was significantly higher in the films complexed with sulfated polysaccharides compared to the BA/HPMC film alone, indicating that these polysaccharides preserved the antioxidant potential of BA during film formation, even under thermal stress. Further evaluation using a Cellular Antioxidant Activity (CAA) assay confirmed that the sulfated polysaccharide-BA films showed higher antioxidant capacity at the cellular level (9.89–13.75 μmol QE equiv./100 mg BA) compared to the control (7.12 μmol QE equiv./100 mg BA). The study found that while CS and FU enhanced cellular uptake and antioxidant efficacy, λ-CG’s higher molecular weight resulted in lower cellular antioxidant activity. Importantly, the oral films demonstrated excellent biosafety, with no adverse effects on cell viability. These findings suggest that sulfated polysaccharides not only stabilize the anthocyanin structure but also enhance its antioxidant properties, making them promising carriers for delivering bioactive compounds in functional oral film applications [147].

Dara Rabêlo Silva et al. investigated the antioxidant potential of two native Brazilian berries, *Eugenia calycina* and *Eugenia stigmatosa*, focusing on their phenolic composition and bioactive properties. The study revealed that both fruits are rich in phenolic compounds, with *E. calycina* showing particularly high levels of anthocyanins, especially cyanidin-3-glucoside (242.97 µg/g), which was strongly correlated with its superior antioxidant capacity. The antioxidant activity was assessed using ORAC, FRAP, and ABTS assays, indicating that anthocyanins play a significant role in the antioxidant efficacy of *E. calycina*. In contrast, *E. stigmatosa* was found to contain higher levels of other phenolics, such as rutin and gallic acid, along with a notable content of condensed tannins, which contributed to its antioxidant profile. The findings highlight the distinct bioactive profiles of the two species, where *E. calycina* stands out for its anthocyanin content, while *E. stigmatosa* offers a rich source of tannins and rutin. The study underscores that these *Eugenia* species, beyond their low caloric value and high dietary fiber content, have potential health benefits linked to their bioactive compounds. The high anthocyanin content in *E. calycina* suggests its strong capacity to counter oxidative stress, which could help prevent chronic diseases. These results provide a basis for further research into the therapeutic applications of these fruits, particularly in exploring how their antioxidant properties might contribute to health when integrated into diets. Moreover, the findings encourage the use of native fruits in promoting healthy eating habits, while also highlighting the importance of conserving Brazilian biodiversity. This study not only supports the value of incorporating these antioxidant-rich fruits into a sustainable diet but also emphasizes their potential to enhance the nutritional quality of functional foods [148].

Sun et al. explored the use of porous gelatin microspheres (PGMs) to enhance the stability and antioxidant capacity of anthocyanins (ANCs), particularly for applications in food preservation and as freshness indicators. The PGMs were created using a calcium carbonate template method, resulting in a highly porous structure that enabled efficient loading of anthocyanins. Through electrostatic interactions at pH 4, the loading capacity reached 353.08 mgACN/gPGMs, significantly enhancing the retention and controlled release of anthocyanins. The study demonstrated that anthocyanin-loaded PGMs (ACN@PGMs) provided prolonged antioxidant activity by delaying the degradation rate of anthocyanins under light and humidity conditions. After 16 days of storage, the degradation rate of free anthocyanins was 56.66%, while that of ACN@PGMs was notably lower at 49.24%, indicating improved stability. This enhanced protection allows the anthocyanins to maintain their antioxidant properties over a longer period. Moreover, ACN@PGMs displayed excellent sensitivity to ammonia, with a visible color shift from purple to yellow, suggesting their potential use as natural indicators of food freshness. These findings underscore the dual functionality of PGMs in protecting anthocyanins’ antioxidant activity while also serving as smart indicators in food packaging, providing a sustainable approach to food quality monitoring [149].

Abdelrahman et al. investigated the antioxidant, antibacterial, and anticancer properties of anthocyanin-rich extracts (AREs) derived from pomegranate peel (*Punica granatum*), chili pepper fruit (*Capsicum annuum*), and bougainvillea flowers (*Bougainvillea spectabilis*). The study aimed to extract and analyze these natural pigments for their bioactive functions using various assays, including DPPH, FRAP, and MTT. The results showed that the ARE from pomegranate peel exhibited the highest total phenolic content (466 mg GAE/g extract) and superior antioxidant activity, followed by bougainvillea flowers and chili pepper fruit. The antioxidant potential of the extracts increased proportionally with concentration, with pomegranate peel demonstrating the strongest free radical scavenging capacity. The antibacterial properties were also notable, with the pomegranate peel extract showing the strongest inhibitory effects against various pathogens [150].

Recently, Kumar Saini et al. conducted a review focused on the latest advancements in the stabilization, bioavailability, and industrial applications of anthocyanins. A key highlight of the review is the emphasis on innovative strategies to enhance the stability of these compounds, given their sensitivity to environmental factors such as pH, light, and temperature. Advanced techniques, including encapsulation and the use of stabilizing agents, are shown to significantly extend the antioxidant efficacy of anthocyanins, making them more suitable for functional food applications. The review also discusses improved bioavailability through the use of nanostructured carriers, which can enhance absorption in the gastrointestinal tract, thereby overcoming one of the major limitations associated with their use. It also explores new methods to utilize the antioxidant properties of an-thocyanins not only in nutraceuticals, but also in innovative applications in the food industry, such as natural freshness indicators and additives. This paves the way for more stable and bioactive formulations in future product development [151].

Given the existence of this recent and comprehensive review by Kumar Saini et al. we will not delve further into this section as their work thoroughly covers the current advances in the stabilization, bioavailability, and applications of anthocyanins. Furthermore, the antioxidant properties of anthocyanins have been thoroughly explored in other recent literature, with Sadowska-Bartosz providing a critical review on the reactivity of anthocyanins and anthocyanidins in various antioxidant assays, highlighting their interactions with reactive oxygen and nitrogen species, metal ions, and other compounds, as well as their effects at cellular and organismal levels [152]. Lakshmikanthan et al. provide a comprehensive review of anthocyanin-rich foods, detailing the extraction and purification techniques for anthocyanins, as well as their medicinal applications, sustainable uses, and potential roles as natural colorants in the food and cosmetic industries [153]. Tena et al. provide a comprehensive review of the sources, bioavailability, and therapeutic effects of anthocyanins, emphasizing factors that influence their antioxidant capacity and the effects of structure, concentration, and environmental conditions on their efficacy [154]. Given the detailed coverage of antioxidant properties in these recent reviews, we will not elaborate on this aspect in our discussion.

### 5.6. Improving Brain Functions

Krikorian et al. (2010) led a human trial which involved a group of nine participants and a placebo group of seven participants close in age and educational level with a decline of learned memory and evaluated their responses at wild blueberry juice. It has been shown that the anthocyanins present in blueberries influenced positively neurocognitive functions and reduced symptoms of depression [110].

### 5.7. Reducing Cardiovascular Diseases

Tian et al. studied the effects of anthocyanins on platelet function in individuals with dyslipidemia. Their findings revealed that anthocyanin supplementation dose-dependently attenuates platelet function, reducing oxidative stress biomarkers and platelet aggregation. Specifically, 12-week supplementation with 80 mg/day of anthocyanins reduced collagen-induced platelet aggregation by 3.39 ± 2.36% and activated glycoprotein GPIIbIIIa by 8.25 ± 2.45% (*p* < 0.05). Supplementation with 320 mg/day of anthocyanins inhibited collagen-induced platelet aggregation by 7.05 ± 2.38%, ADP-induced platelet aggregation by 7.14 ± 2.00%, reduced platelet ROS levels by 14.55 ± 1.86%, and improved mitochondrial membrane potential by 7.40 ± 1.56% (*p* < 0.05). These results indicate that anthocyanins can effectively reduce platelet function in individuals with dyslipidemia, thereby contributing to cardiovascular health [155].

### 5.8. Skin-Protective Properties

Correia et al. studied the effects of anthocyanins on skin protection. The study found that anthocyanins, specifically cyanidin-3-*O*-glucoside and malvidin-3-*O*-glucoside, exhibit significant skin protective properties. These compounds were shown to reduce biofilm production by *S. aureus* and *P. aeruginosa*, attenuate reactive oxygen species (ROS) production in human skin keratinocytes and fibroblasts and inhibit skin-degrading enzymes. Notably, anthocyanins demonstrated UV-filter capacity, with SPF values ranging from approximately 14 to 30. Carboxypyranocyanidin-3-*O*-glucoside stood out for its overall performance, suggesting potential for incorporation in topical formulations to enhance skin health [156].

### 5.9. Anticancer

Their potential antitumor effects are attributed to a diverse range of biological activities, including antioxidant and anti-inflammatory properties; anti-mutagenesis; induction of differentiation; inhibition of proliferation through modulation of signal transduction pathways; induction of cell cycle arrest; stimulation of apoptosis or autophagy in cancer cells; and inhibition of invasion and metastasis [105].

Regarding the anti-mutagenic effects of anthocyanins, investigated the anti-mutation effects of four different kinds of sweet potato roots using Salmonella typhimurium TA98. They found that these sweet potato roots, containing 3-(6,6′-caffeylferulylsophoroside)-5-glucoside of cyanidin (YGM-3) and 3-(6,6′-caffeylferulylsophoroside)-5-glucoside of peonidin (YGM-6), inhibited the reverse mutation of TA98 in a dose-dependent manner. Similarly, the water extracts prepared from the storage roots of four sweet potato varieties with different flesh colors were investigated for their antimutagenic effects. The extract from the whole roots of the purple-colored Ayamurasaki variety effectively decreased the reverse mutation induced by various mutagens, including Trp-P-1, Trp-P-2, IQ, Bla]P, 4-NQO, and dimethyl sulfoxide extracts of grilled beef. Comparison of the inhibitory activity of extracts from normal Ayamurasaki and its anthocyanin-deficient mutant suggested that anthocyanin pigments in the flesh decrease the mutagenic activity of heterocyclic amines. Two anthocyanin pigments purified from purple-colored sweet potato, YGM-3 and YGM-6, effectively inhibited the reverse mutation induced by heterocyclic amines, Trp-P-1, Trp-P-2, and IQ, in the presence of rat liver microsomal activation systems. Therefore, it was concluded that YGM-3 and YGM-6 could inhibit the reverse mutation of normal cells induced by mutagens. Given that oxidative stress from free radical abnormalities can lead to DNA injury and gene mutations—resulting in carcinogenesis—anthocyanins with antioxidant properties may protect human cells from malignant mutations caused by extreme levels of ROS and free radicals, thereby exerting their anti-mutagenesis effects in human somatic cells [157].

Chang Hui et al. studied the anticancer effects of an anthocyanin-rich extract from black rice (AEBR) on breast cancer cells both in vitro and in vivo. Their findings demonstrated that AEBR significantly reduced the viability of various breast cancer cell lines, including MCF-7 (ER+, HER2/neu−), MDA-MB-231 (ER−, HER2/neu−), and MDA-MB-453 (ER−, HER2/neu+). AEBR induced apoptosis in MDA-MB-453 cells via the intrinsic pathway by activating the caspase cascade, cleaving poly (ADP-ribose) polymerase (PARP), depolarizing the mitochondrial membrane potential, and releasing cytochrome C. In vivo, oral administration of AEBR (100 mg/kg/day) to BALB/c nude mice bearing MDA-MB-453 cell xenografts significantly suppressed tumor growth and angiogenesis. This suppression was achieved by downregulating the expression of key angiogenesis factors, including MMP-9, MMP-2, and uPA in tumor tissue. These results suggest that AEBR exerts its anticancer effects against human breast cancer cells by inducing apoptosis and inhibiting angiogenesis [158].

## 6. Applications

### 6.1. Anthocyanins

#### 6.1.1. Food Application

Giusti M. et al. successfully investigated that red radish anthocyanin extract could be an alternative for coloring maraschino cherries substituting allura red. They also examined if anthocyanins obtained from red radish and red-flashed potato had similar coloring properties as allura red in beverages, red radish anthocyanins showing higher stability during storage. Also, for dairy products red radish and carrot anthocyanins used together or separately could provide a red color in these systems as red cabbage gave a purple color [159].

Maner S. et al. achieved a study for cookies manufacturing in which they shifted wheat flour with wine grape pomace powder at four concentrations 5, 10, 15, and 20%, which led to an increased content of anthocyanins in final products (2.03–3.51 mg/g for samples with extract compared with 0.16 mg/g for the control sample). It has been shown that the products which were obtained by adding this powder had enhanced antioxidant properties, more nutrients, and a more intense color [160].

López C.J. et al. found a possible use for *Arbustus unedo* L. extract, which is rich in anthocyanins (especially cyanidin-3-*O*-glucoside), for wafers improving antioxidant activity and providing a more attractive color in these products [161].

In a study, Albuquerque B.R. et al. (2020) used jaboticaba epicarp extract to obtain anthocyanin-based colorant and successfully gave the macarons a stable color [162].

#### 6.1.2. Preparation and Characterization of Anthocyanin-Enriched Bio-Based Films

Chayavanich K. et al. developed gelatine and starch-based films incorporated with red radish anthocyanins and successfully observed seafood and chicken meat spoilage. In another study, Jiang G. et al. prepared a film based on carboxymetil-cellulose-starch and anthocyanins from sweet potato to monitor the freshness of fish. The films were sensitive to ammonia and had a pink color at the initial moment, a purple color after 24 h and a bluish violet after 48 h of storage at 5 °C. For monitoring salmon’s freshness, Xiaowei H. et al. incorporated anthocyanins extracted from black wolfberry, roselle, morning glory, purple potato, rose, carnation, mulberry, red cabbage, and grapes in agar-PVA films [12,163,164].

Zhai X. et al. developed biodegradable films using gelatine, gellan gum and red radish to use them as gas sensors to detect milk and fish spoilage. They observed color changes from orange to yellow in the pH range from 2 to 12 [165].

Mustafa P. et al. investigated if films based on PVA-starch incorporated with 10% and 20% propolis extracts can detect and protect pasteurized milk’s spoilage. After being applied for 48 h on milk samples, the films with 10% propolis extract changed their color from brown to reddish pink and also the films containing 20% propolis extract showed antibacterial activity against gram-negative and gram-positive bacteria (*E. coli* and MRSA) [166].

Pirsa S. et al. developed antibacterial and biodegradable films using chitosan, pomegranate peel extract and *Mellisa officinalis* Essences in different quantities and studied if they can be used as detectors of pH changes for cream cheese. The results showed that for detecting spoilage in cream cheese the chitosan-based films with 0.03% extract had a good sensitivity at pH changes. Regarding the antibacterial activity it has been shown that films containing essence in addition to the extract had antimicrobial activity against gram-positive bacteria (*B. cereus*) [167].

Mohammadalinejhad S. et al. extracted anthocyanins from *Echium amoenum* flowers, incorporated them in bacterial cellulose films and investigated if they can detect pH changes of shrimps. They observed color changes in the range of pH from 2 to 12 from violet to grey and yellow after 4 days at 4 °C [168].

#### 6.1.3. Cosmetics

Saffron (*Crocus sativus*) presents antioxidant and anti-inflammatory properties due to the presence of carotenoids, phenolics, and flavonoids. Due to these properties, Akhtar et al. developed a cream formulation containing saffron extracts with significant depigmentation and antierytema effects [169]. Golmohammadzadeh et al. successfully prepared and tested nanoliposomes containing safranal as natural sunscreen factor [170].

Extracts of stems, leaves, and pomace of sour cherry (*Prunus Cerasus*) were used by Maurícío E. et al. as ingredients in beauty products. They led a human trial on 10 volunteers and tested the protective effects of the antioxidant extracts against methyl nicotinate (an irritation substance) explaining the antioxidant activity from in vitro methods [171,172]. Sim Y. et al. developed a cosmetic formulation containing kenaf (*Hibiscus cannabinus* L.) leaves extract, evaluated its antioxidant, anti-aging, and antimelanogenic activities and determined the microbiological properties, storage stability, and in vitro cytotoxicity. They concluded that kenaf leaves are a valuable ingredient with skincare benefits for the cosmetic industry [173].

Inman C. et al. proposed an application for black bean (*Phaseolus vulgaris*) as natural colorant. They developed an efficient semi-permanent hair coloring gel which is consistent with use in industrial practice [174].

Jati leaves extract (*Tectona grandis*) was used by Setyawaty R. et al. as natural dye for coloring lipsticks. The lipstick obtained had no change of color, odor, and shape after 30 days of storage [175]. Lwin et al. used red dragon fruit (*Hylocereus polyrhizus*), which contains betacyanin pigment, in a natural lipstick. They evaluated the pH, melting point, aging stability, and antioxidant stability as qualitative parameters. Regarding the safety, they determined the lead content, carried out a skin irritation test, and a microbial analysis [176].

In another study, Lourith et al. successfully used the butterfly pea (*Clitoria ternatea*) anthocyanins microencapsulation to develop a rouge based on cornstarch and additional ingredients [177].

The coloring and antioxidant properties of anthocyanins and their derivatives have also been utilized in hair dyes and cosmetic dermatology products [178,179].

Giusti et al. demonstrated that anthocyanins incorporated into lipstick formulations retain their antioxidant properties, effectively scavenging free radicals and acting as UV filters. This effect is particularly pronounced in anthocyanins acylated with cinnamic acid [180].

### 6.2. Flavylium Dyes

#### 6.2.1. Applications in Analytical Chemistry

Monitoring metals and metal ions represents a major task, specifically in environmental or biological samples. Flavylium-inspired compounds were proved as useful compounds for their fast and sensitive detection and quantitation in aqueous media. Cloud point extraction (CPE) emerged as a promising analytical technique for separation and preconcentration of trace elements, particularly metals. Snigur et al. developed a rapid CPE method for preconcentration and subsequent spectrophotometric determination of Molybdenum (VI) using a synthetic flavylium derivative, 6,7-dihydroxy-2,4-diphenylbenzopyrylium perchlorate, for extraction (as a red complex) at room temperature into a chemically induced Triton X-100 surfactant-rich phase, and measurement of absorbance at 560 nm. The calibration plot was linear in the Molybdenum (VI) concentrations range of 7.9–160 μg/L and the detection limit was 2.3 μg/L [181]. Similarly, 6,7-dihydroxy-2,4-diphenylbenzopyrylium chloride was successfully used for room-temperature CPE of Copper (II), generating an extractable complex in weekly acidic medium. The optimal conditions were pH 5.0, 1% Triton X-100 (*v*/*v*), and 3.75 × 10^–2^ M sodium salt of p-toluic acid (chemical initiator). The linear calibration domain and the limit of detection were 6–870 μg/L and 6 μg/L Copper (II) concentrations, respectively [182].

Fluorescent chemosensors represent another application direction of flavylium-derived compounds for analytical purposes. Mercury (II) ion detection was accomplished by Cheng et al. who developed a “turn-on” fluorescent probe based on a flavylium-derived scaffold, able to detect Hg^2+^ with high sensitivity (limit of detection 0.47 nM). The method was applicable not only in water but also in urine samples and in living cells [183]. This research was developed based on previous reports, such as that of Ren et al. which demonstrated the possibility of designing new fluorescent materials, inspired by anthocyanidins, with flexibly tunable emission spectra covering the whole visible light range, from 467 to 707 nm [184].

Gomes et al. reported a Near InfraRed (NIR) emissive fluorescent chemosensor for Zn^2+^ and other divalent metal ions detection, based on a styrylflavylium dye with a di-(2-picolyl)amine moiety as the metal chelating unit [185].

The real-time detection and monitorization of H_2_S is also important in water, food, and living cells. Wang et al. designed an asymmetrical flavylium-based probe with large Stokes shift of 81 nm and NIR emission at 671 nm for H_2_S detection. Linear correlation was observed for H_2_S concentrations from 0 to 50 μM, with limit of detection of 0.31 μM [186].

#### 6.2.2. Color Indicator Sensors

Galindo F. et al. encapsulated a fluorescent flavylium salt in a water permeable cross-linked poly(2-hydroxyethyl methacrylate) polymer matrices to determine humidity and ammonia content of the surrounding atmosphere of the films [187].

Gomes V. et al. immobilized in the presence and absence of glycerol in a cellulose acetate matrix a pyranoflavylium dye and obtained films whose response was monitored at different pH values ranging from 4 to 8 and in the atmosphere of solutions containing ammonia at different concentrations and in the presence of biogenic amines [188].

In another study, Gomes V. et al. immobilized a pyranoflavylium dye and glycerol into a cellulose acetate matrix and applied the obtained film to monitor meat and fish freshness. The detection of fish spoilage in 5 days of storage is shown to be very effective, with films’ color changing from yellow to purple [189].

#### 6.2.3. Topical Photodynamic Therapy

Oliveira H. et al. studied five amino-based flavylium compounds as photosensitizers in topical photodynamic therapy (PDT) which is an alternative to surgery for several infectious skin diseases and skin cancers and three of them were suitable to be used in PDT [19].

## 7. Conclusions and Future Perspectives

The isolation of anthocyanins and the synthesis of bio-inspired flavylium compounds have significantly advanced our understanding of these bioactive molecules and their applications. Various extraction techniques, such as solvent extraction, microwave-assisted extraction, and supercritical fluid extraction, have been optimized to yield high-quality anthocyanins from natural sources. Indicating the best extraction method is challenging, since the choice of a particular technique is influenced by several factors such as the anthocyanins content in the tissue of the selected plant source, the possible degradation during the extraction process or the overall costs of process and equipments. Solvent extraction is still regarded as the most convenient method, using mainly water combined with water-miscible organic solvents at relatively moderate temperatures and reduced equipment costs compared to other methods, but the is hampered by the lower extraction efficiency and the possible toxicity of the organic co-solvent. The emerging techniques developed in the last years, ultrasound, microwave, and supercritical CO_2_ assisted extraction, provide higher extraction yield and shorter extraction time but involve higher equipment costs and the mechanical or thermal effects generated during the process can affect the structure of the anthocyanins. Supercritical CO_2_ assisted extraction is in our opinion the most promising extraction method avoiding the use of organic solvents and the degradation of the product due to severe extraction conditions. However, the production costs are high; therefore, the enzyme-assisted extraction could be developed in the near future as a valuable alternative, leading to high extraction yields in mild conditions and reducing the amount or even eliminating the use of an organic solvent.

Additionally, chemical synthesis pathways for flavylium derivatives have opened new directions for the development of synthetic bioactive compounds with tailored properties. Nowadays, the existing synthetic methods allow the preparation of a wide variety of flavylium derivatives with different substitution paths, which can provide them pharmacological effectiveness. We presume that in this respect the development will be continuous and spectacular.

Analytical methods, including chromatography, spectroscopy, and mass spectrometry, have proven essential for the detailed characterization of these compounds, ensuring their purity and stability. The anthocyanins obtained from plants are usually concentrated extracts which can be utilized without advanced separation and purification, but their composition must be known. For the identification of the anthocyanins present in a mixture hyphenated techniques, particularly HPLC-MS are the most useful. Although not being just as accurate, UV-Vis spectroscopy is a more accessible technique which can provide valuable information concerning the structural composition of an anthocyanin mixture, particularly the presence of certain functional groups. Considering the synthetic flavylium derivatives, 2D-NMR is the best analytical method to demonstrate the exact structure of a compound.

Studies on the preventive and pharmacological effects of flavylium compounds suggest promising therapeutic potentials, including antioxidant, anti-inflammatory, and anticancer activities. The antioxidant and anti-inflammatory effects are well documented and already used in diverse preparations, the so-called food supplements, which are out of the scope of this review. It should be pointed out that in our opinion their main benefit is rather preventive than curative. Other pharmacological effects, particularly the anti-cancer activity is the object of intense studies; however, it must be mentioned that is a long way between the in vitro studies on cancer cell lines and the clinical trials demonstrating the efficiency of a specific compound. Certainly, this direction will represent a major objective in the near future and inhibition of proliferation of certain cancer cells by natural or synthetic flavylium derivatives might have a clear perspective, specifically in the development stage. In terms of food applications, anthocyanins continue to be explored as natural dyes and functional ingredients, contributing not only to the aesthetic appeal of food products but also offering health benefits. Looking forward, further research is needed to refine extraction and synthesis methods, enhance the bioavailability of these compounds, and explore novel applications in food, pharmaceuticals, and other industries, thereby expanding their commercial potential and therapeutic scope.

## Figures and Tables

**Table 1:** The most important anthocyanins found in different plant sources.

Source	Main Anthocyanins	Reference
Black wolfberry (*Lycium ruthenicum*)	cyanidin-3-glucoside, petunidin-3-p-coumaroyl-5-glucoside, petunidin-3-*O*-rutinoside(trans-p-coumaroyl)-5-*O*-glucoside	[12]
Roselle (*Hibiscus sabdariffa*)	delphinidin-3-*O*-sambubioside	[12]
Morning glory (*Ipomoea purpurea*)	peonidin-3-sophoroside-5-glucoside	[12]
Purple potato (*Solanum tuberosum* L.)	petunidin-3-p-coumaroyl-5-glucoside, cyanidin-3-myrosinate-glucoside, cyanidin-3,5 glucoside	[12]
Carnation (*Dianthus caryophyllus*)	cyanidin-3-*O*-glucoside	[12]
Mulberry (*Morus rubra*)	pelargonidin-3-*O*-glucoside, cyanidin-3-rutinosides	[12]
Red cabbage (*Brassica oleracea*)	cyanidin-3-diglcoside-5-glcoside, pelargonidin-3-sinapoyl -diglucoside-5-glucoside	[12]
Grapes (*Vitis vinifera L.*)	malvidin-3-*O*-glucoside, cyanidin-3-*O*-glucoside	[12]
Maize (*Zea mays*)	cyanidin-3-*O*-glucoside, cyanidin-3-*O*-succinylglucoside	[13]
Cowpea (*Vigna unguiculata*)	delphinidin-3-O-glucoside, cyanidin-3-O-glucoside	[13]
Strawberry (*Fragaria ananassa*)	cyanidin-3-*O*-glucoside	[14]
Apple (*Malus pumila* L.)	cyanidin-3-galactoside	[15]
Apricot (*Prunus armeniaca* L.)	cyanidin-3-rutinoside	[15]
Cherry (*Prunus avium* L.)	cyanidin-3-glucoside, cyanidin-3-rutinoside	[15]
Ginger (*Zingiber officinale* *Roscoe*)	cyanidin-3-glucoside, peonidin-3-rutinoside	[15]
Jambul (*Syzygium cumini* L.)	delphinidin-3,5-diglucoside, petunidin-3,5-diglucoside, malvidin-3,5-diglucoside, peonidin-3,5-diglucoside and cyanidin -3,5- diglucoside	[15]
Grosella (*Ribes rubrum* L.)	cyanidin-3-xylosylrutinoside, cyanidin-3-glucosylrutinoside, cyanidin-3-sambubioside, cyanidin-3-rutinoside, cyanidin-3- glucoside	[15]

**Table 2:** Selected examples of synthetic flavylium derivatives with 2-phenyl-1-benzopyrilium structural units, reported in the literature.

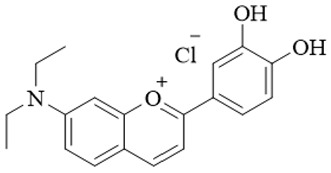	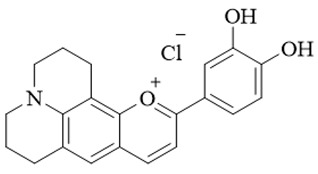
Amino-substituted flavylium derivatives [18]	
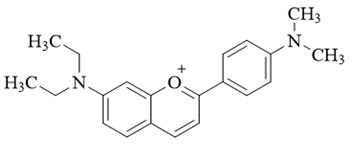	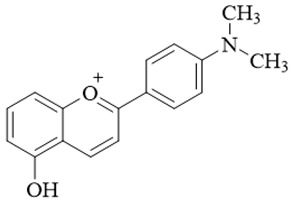
Amino-substituted flavylium derivatives (7-diethylamino-4′-dimethylamino-benzopyrylium, 5-hydroxy-4′-(dimethylamino)-benzopyrylium) [19]	
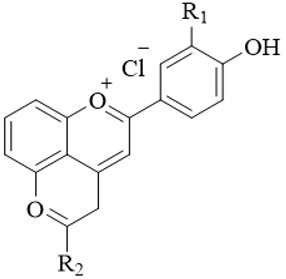	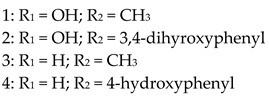
Pyrano-3,7-deoxyanthocyanin derivatives [17]	
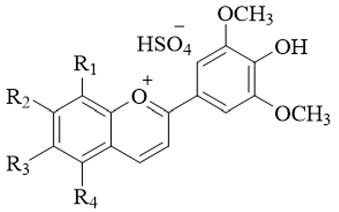	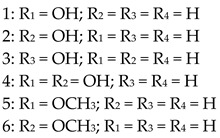
Flavylium derivatives with different methoxy and hydroxy substitutions in ring **A** [20]	
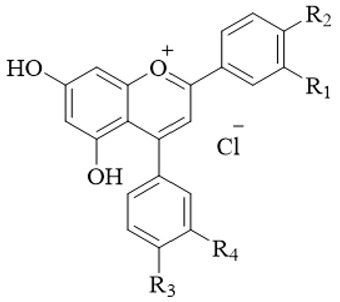	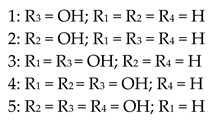
Flavylium derivatives 4-substituted with a mono-, di-, or trihydroxyphenyl functional group [7]	
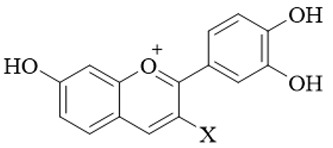	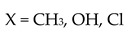
3’,4’,7-Trihydroxy flavylium derivatives with different substituents in position 3 [21]	

**Table 3:** Extraction conditions applied for the recovery of anthocyanins from different matrices.

Source of Anthocyanins	Solvent	Temperature	Anthocyanins Concentration in the Extracted Product	Source
plum peels	citric acid:ethanol—80:20	40 °C	105.5 mg/kg	[22]
dry blueberry pomace	Water, supplemented with 1% *w*/*v* citric acid	80 °C	19,440.7 mg/kg	[23]
red radish peels	50% ethanol and acidified water	25 ± 2 °C	372.6 mg/L	[24]
whole blueberry	70% acetone, 0.01% HCl, and chloroform	25 °C	364.1 mg/L	[25]
whole blueberry	methanol and 0.01% citric acid	25 °C	627.2 mg/L	
blueberry pomace	70% acetone, 0.01% HCl, and chloroform	25 °C	982.2 mg/L	
blueberry pomace	80% ethanol	25 °C	813.7 mg/L	
jabuticaba skins	water, ethanol, and citric acid	25 °C	32.06 mg/L	[26]
jabuticaba skins	water, methanol, and citric acid	25 °C	36.40 mg/L	
cabbage leaves	water, ethanol, and adipic acid	25 °C	53.82 mg/L	
cabbage leaves	water, methanol, and adipic acid	25 °C	56.80 mg/L	
eggplant peels	water, ethanol, and citric acid	60 °C	19.75 g/kg	[27]
black chokeberry fruits	Water:glycerol—1:1	20 °C, 50 °C, 80 °C	70.02–92.62 mg/L	[28]
black chokeberry fruits	Water:glycerol—2.75:1	20 °C, 50 °C, 80 °C	55.90–82.98 mg/L	
elderberry fruits	Water:glycerol—11.91:1	20 °C, 50 °C, 80 °C	33.56–34.88 mg/L	
elderberry fruits	Water:glycerol—1:1	20 °C, 50 °C, 80 °C	31.99–34.33 mg/L	
elderberry fruits	Water:glycerol—2.75:1	20 °C, 50 °C, 80 °C	30.01–35.59 mg/L	
borage flowers	choline chloride and glycerol-based	25 °C	10.16–1798.48 mg/kg	[29]
mangosteen peel	(K_2_CO_3_)-glycerol—1:7	25 °C	263.976 mg/L	[30]

**Table 4:** Conditions applied for microwave-assisted extraction of anthocyanins from different matrices.

Source of Anthocyanins	Microwave Power [W]	Temperature [°C]	Time [min]	Anthocyanins Amount	Reference
Red raspberries (*Rubus idaeus* L.)	366	25	12.1	43.42 mg/100 g	[38]
Sweet cherries	1000	115	3	30 mg/100 g cyanidin-3-*O*-glucoside and 60 mg/100 gcyanidin-3-*O*-rutinoside	[39]
Chinese bayberry	800	80	15	2.95 ± 0.08 mg/g	[40]
Cocoa shell	100	-	2	11.85 × 10^−4^ M	[41]
Purple sweet potatoes (*Ipomoea batatas* L.)	270	80	3.13	311.64 mg/L	[42]
Blueberry peels	800	60	30	634.71 mg/100 g	[43]

**Table 6:** Conditions applied for supercritical CO_2_ assisted extraction of anthocyanins from different matrices.

Source of Anthocyanins	Temperature	Flow Rate	Water Pressure	Extracted Anthocyanins	Source
Roselle (*Hibiscus sabdariffa*)	First step: 40 °C Second step: 140 °C	First step: 4.875 mL/min Second stage: 8 mL/min	First step: 20 MPa Second step: 12 MPa	1142.61 mg/100 g	[57]
Eggplant peel	60 °C	2 L/min	10 MPa	17.04 g/kg	[27]
Haskap berry pulp	65 °C	10 mL/min	45 MPa	^a^ 52.7%	[58]

^a^ Total anthocyanins (TA) yield (mg TA in the extract compared with mg TA in the initial biomass).

**Table 7:** HPLC analysis conditions for anthocyanins from different matrices.

Source of Anthocyanins	Anthocyanin Types	Eluent System	Detector	Wavelength [nm]	HPLC Column	Reference
*Ficus padana burm* L.	pelargonidin3-(6”-p-coumarylglucoside)-5-(4”’-malonylglucoside) pelargonidin 3-(6′’-malonylglucoside)	A: formic acid solution B: acetonitrile: water: formic acid (49:49:2)	DAD-ESI-MS	516	Shimpack (Kyoto, Japan) PRC-ODS (20 × 250 mm, 5 μm)	[72]
Black rice (*Oryza sativa* L. *indica*)	cyanidin-3-glucoside peonidin-3-d-glucoside	A: milli-Q-water B: methanol: formic acid (20:5)	UV *	530	C18 (4.6 × 250 mm, 5 μm)	[73]
Cranberry pomace	cyanidin 3-galactoside cyanidin 3-arabinoside peonidin 3-galactoside peonidin 3-arabinoside	A: water: phosphoric acid (99.5:5) B: water: acetonitrile: glacial acetic acid (50:48.5:0.5)	UV *	520	5C18-PAQ (4.6 × 150 mm, 10 μm)	[74]
Haskap berry	cyanidin-3-glucoside cyanidin-3-rutinoside pelargonidin-3-glucoside peonidin-3-*O*-glucoside	A: water: methanol (90:10) with formic acid B: methanol with formic acid	DAD *	520	Max-RP C12 (4.6 × 250 mm, 4 μm)	[58]
Pomegranate (*Punica granatum* L.)	delphinidin-3,5-diglucoside cyanidin-3,5-diglucoside delphinidin-3-glucoside pelargonidin-3,5-diglucoside cyanidin-3-glucoside pelargonidin-3-glucoside	A: formic acid solution B: acetonitrile	PDA ** and ESI-QTOF MS-MS	520	Thermo Scientific (Massachusetts, USA) C18 BDS (4.6 mm × 100 mm, 2.4 μm)	[75]
Blackcurrants (*Ribes nigrum*)	cyanidin-3-rutinoside cyanidin delphinidin-3-rutinosides delphinidin	A: H_3_PO_4_ in water B: acetonitrile	UV-MS	520	Lichrospher 100 RP C18 (4 mm ×125 mm, 5 μm)	[76]
Purple corn (*Zea mays L.*)	cyanidin-3-glucoside pelargonidin-3-glucoside peonidin-3-glucoside cyanidin-3-(6-malon)-glucoside pelargonidin-3-(6-malon)-glucoside peonidin-3-(6-malon)-glucoside	A: trifluoroacetic acid in water B: acetonitrile	PDA-MS	520	Lichrospher C-18 (2.1 mm × 250 mm, 5 μm)	[77]
Blueberry Pomace	cyanidin-3-glucoside delphinidin-3-arabinosidechloride petunia-3-glucoside petunia-3-arabinoside peony-3-glucoside malvidin-3-glucoside delphinidin-3-glucoside	A: formic acid solution B: formic acid in acetonitrile	ESI-MS/MS	520	Agilent (Santa Clara, California USA) ZORBAX SB-C18 (4.6 × 250 mm, 5 μm)	[34]
Butterfly pea (*Clitoria ternatea)*	delphinidin-3-(6′′ -p-coumaroyl)-rutinoside cyanidin 3-(6′′ -p-coumaroyl)-rutinoside delphinidin-3-(cis-p-coumaroyl-glucoside) delphinidin-3-(trans-p-coumaroyl-glucoside) cyanidin-3-(p-coumaroyl) glucose delphinidin-3-pyranoside	A: formic acid solution B: formic acid in acetonitrile	UV-MS	535	Zorbax Eclipse C18 (2.1 × 50 mm, 1.8 μm)	[78]

* Standards were used for identification and quantification; ** Single isolated anthocyanin standards were used for quantitation.

**Table 8:** Analysis of anthocyanins extracted from different food matrices by MALDI-TOF MS.

Sample	Identified Anhocyanins	Matrix	Reference
Red wine	peonidin 3-glucoside delphinidin 3-glucoside petunidin 3-glucoside malvidin 3-glucoside malvidin 3-glucosideacetate peonidin 3-glucoside-coumarate petunidin 3-glucoside-coumarate malvidin 3-glucoside-coumarate malvidin 3-glucoside-caffeoate	2,4,6-Trihydroxyacetophenone (THAP)	[89]
Concord grape juice	3-glucoside peonidin 3-glucoside delphinidin 3-glucoside petunidin 3-glucoside malvidin 3-glucoside cyanidin 3-glucoside-coumarate peonidin 3-glucoside-coumarate cyanidin 3,5-diglucoside delphinidin 3-glucoside-coumarate peonidin 3,5-diglucoside petunidin 3-glucoside-coumarate malvidin 3-glucoside-coumarate cyanidin 3-glucoside-coumarate-5-glucoside peonidin 3-glucoside-coumarate-5-glucoside petunidin 3-glucoside-coumarate-5-glucoside malvidin 3-glucosidecoumarate-5-glucoside		
Raspberry syrup	cyanidin 3-glucoside cyanidin 3-rutinoside pelargonidin 3-sophoroside cyanidin 3-sophoroside cyanidin 3-(2G-glucosylrutinoside)		
Cranberry juice	cyanidin 3-arabinoside peonidin 3-arabinoside cyanidin 3-galactoside peonidin 3-galactoside		
Strawberry	pelargonidin-3-*O*-glucoside cyanidin-3-*O*-glucoside delphinidin-3-*O*-glycoside pelargonidin-3-*O*-malonylglucoside cyanidin-3-*O*-malonylglucoside pelargonidin-3-*O*-rutinoside	2,5-Dihydroxybenzoic acid	[91]
Red grapes	peonidin malvidin peonidin-3-glucoside petunidin-3-glucoside malvidin-3-glucoside peonidin-3-glucoside-p-coumarate peonidin-3,5-diglucoside malvidin-3-glucoside-p-coumarate	α-Cyano-4-hydroxycinnamic acid (CHCA)	[92]
Plum	cyanidin cyanidin-3-glucoside cyanidin-3-glucoside-p-coumarate cyanidin-3-sophoroside	α-Cyano-4-hydroxycinnamic acid	
Capulin (*Prunus salicifolia*)	cyanidin-3-glucoside [cyanidin-3-glucoside deprotonated]K^+^ cyanidin-3-glucoside-p-coumarate [cyanidin-3-rutinoside deprotonated]K^+^	α-Cyano-4-hydroxycinnamic acid	
Blue corn	cyanidin-3-glucoside-p-coumarate peonidin-3-glucoside cyanidin-3-glucoside pelargonidin-3-glucoside pelargonidin	CHCA	[93]

**Table 9:** Comparison of calculated and measured absorption wavelengths (λ, in nm) for various flavylium derivatives [20].

Compound		Calculated Absorption Wavelength λ (nm)		Measured Absorption Wavelength λ (nm)	
1		482.1		471	
2		456.6		482	
3		465.1		490	
4		476.3		497	
5		474.7		472	
6		462.4		480	
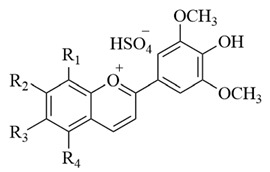			1: R1=OH; R2=R3=R4=H 2: R2=OH; R1=R3=R4=H 3: R3=OH; R1=R2=R4=H 4: R1=R2=OH; R3=R4=H 5: R1=OCH3; R2=R3=R4=H 6: R2=OCH3; R1=R3=R4=H		

## Data Availability

No new data were created or analyzed in this study.
